# Diselenophene‐Dithioalkylthiophene Based Quinoidal Small Molecules for Ambipolar Organic Field Effect Transistors

**DOI:** 10.1002/advs.202305361

**Published:** 2023-12-14

**Authors:** Arulmozhi Velusamy, Yen‐Yu Chen, Meng‐Hao Lin, Shakil N. Afraj, Jia‐Hao Liu, Ming‐Chou Chen, Cheng‐Liang Liu

**Affiliations:** ^1^ Department of Chemistry and Research Center of New Generation Light Driven Photovoltaic Modules National Central University Taoyuan 32001 Taiwan; ^2^ Department of Materials Science and Engineering National Taiwan University Taipei 10617 Taiwan

**Keywords:** ambipolar, organic transistors, quinoid, selenophene, solution‐processing

## Abstract

This work presents a series of novel quinoidal organic semiconductors based on diselenophene‐dithioalkylthiophene (**DSpDST**) conjugated cores with various side‐chain lengths (‐thiohexyl, ‐thiodecyl, and ‐thiotetradecyl, designated **DSpDSTQ‐6**, **DSpDSTQ‐10**, and **DSpDSTQ‐14**, respectively). The purpose of this research is to develop solution‐processable organic semiconductors using dicyanomethylene end‐capped organic small molecules for organic field effect transistors (OFETs) application. The physical, electrochemical, and electrical properties of these new **DSpDSTQ**s are systematically studied, along with their performance in OFETs and thin film morphologies. Additionally, the molecular structures of **DSpDSTQ** are determined through density functional theory (DFT) calculations and single‐crystal X‐ray diffraction analysis. The results reveal the presence of intramolecular S (alkyl)···Se (selenophene) interactions, which result in a planar SR‐containing **DSpDSTQ** core, thereby promoting extended *π*‐orbital interactions and efficient charge transport in the OFETs. Moreover, the influence of thioalkyl side chain length on surface morphologies and microstructures is investigated. Remarkably, the compound with the shortest thioalkyl chain, **DSpDSTQ‐6**, demonstrates ambipolar carrier transport with the highest electron and hole mobilities of 0.334 and 0.463 cm^2^ V^−1^ s^−1^, respectively. These findings highlight the excellence of ambipolar characteristics of solution‐processable OFETs based on **DSpDSTQ**s even under ambient conditions.

## Graphical abstract

A series of novel quinoidal organic semiconductors based on diselenophene‐dithioalkylthiophene (**DSpDST**) conjugated cores with various side‐chain lengths are designed for solution‐processable organic field effect transistors (OFETs). Remarkably, the compound with the shortest thioalkyl chain, **DSpDSTQ‐6**, demonstrates ambipolar carrier transport with the highest electron and hole mobilities of 0.334 and 0.463 cm^2^ V^−1^ s^−1^, respectively.

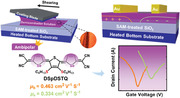

## 1 Introduction

The pursuit of air‐stable, solution‐processable organic semiconductors has garnered significant attention in the development of high‐performance organic field effect transistors (OFETs) for a wide range of applications, including memory devices, smart cards, radio‐frequency identification tags, transparent circuits, electronic papers, flexible displays, and sensors.^[^ ^1^, ^2^, ^3^, ^4^, ^5^, ^6^, ^7^, ^8^, ^9^, ^10^, ^11^, ^12^, ^13^, ^14^ ^]^ These devices offer attractive features such as desirable electronic properties, flexibility, cost, versatile functionalization, and processability. Among the various parameters defining OFET performance, the charge‐carrier mobility (*μ*) is a critical factor that quantifies the average drift velocity of the charge carriers (holes or electrons) per unit electric field. The realization of high‐performance OFETs necessitates the utilization of organic semiconductor materials with high charge carrier mobility, good environmental stability, and ease of processability.^[^ ^15^, ^16^, ^17^, ^18^, ^19^, ^20^ ^]^ Such novel semiconductor materials can be obtained via advancements in material science and device fabrication techniques.^[^ ^21^, ^22^ ^]^ In particular, thiophene‐based compounds such as oligothiophenes and fused thiophene aromatics have demonstrated exceptional performance in this regard.^[^ ^23^, ^24^, ^25^, ^26^, ^27^, ^28^, ^29^ ^]^ Notably, these organic semiconductors rely on weak intermolecular interactions, such as Van der Waals, S–S, *π–π*, and C–H interactions, rather than the strong covalent bonds present in inorganic semiconductors.^[^ ^30^ ^]^ Consequently, enhancing these intermolecular interactions emerges as a promising avenue for improving the OFET performance.

Dicyanomethylene (DCN)‐end‐capped quinoidal structures have emerged as a promising platform for organic semiconductors due to their unique electronic properties, which can be attributed to several factors. Firstly, the presence of *π*‐electron conjugation along the alternating C =  C/C− C bonds of the quinoid backbone leads to enhanced delocalization,^[^ ^31^ ^]^ and this intrinsic aromaticity provides a relatively low reorganization energy compared to that of other aromatic compounds.^[^ ^32^ ^]^ In addition, the potent alkyl‐acceptor moieties of the quinoidal compounds create a highly hybridized frontier molecular orbital system, thus leading to a deep lowest unoccupied molecular orbital (LUMO) and an enhanced highest occupied molecular orbital (HOMO). This energy level modulation favors the development of high‐mobility donor‐acceptor compounds based on a variety of donors and acceptors that exhibit effective charge carrier transport.^[^ ^33^ ^]^ Thus, the addition of two strongly electron‐withdrawing groups (DCN) affords low‐lying LUMO energy levels, thereby leading to facile electron injection and improving the ambient stability of the OFET.^[^ ^34^, ^35^ ^]^

**Figure 1:**
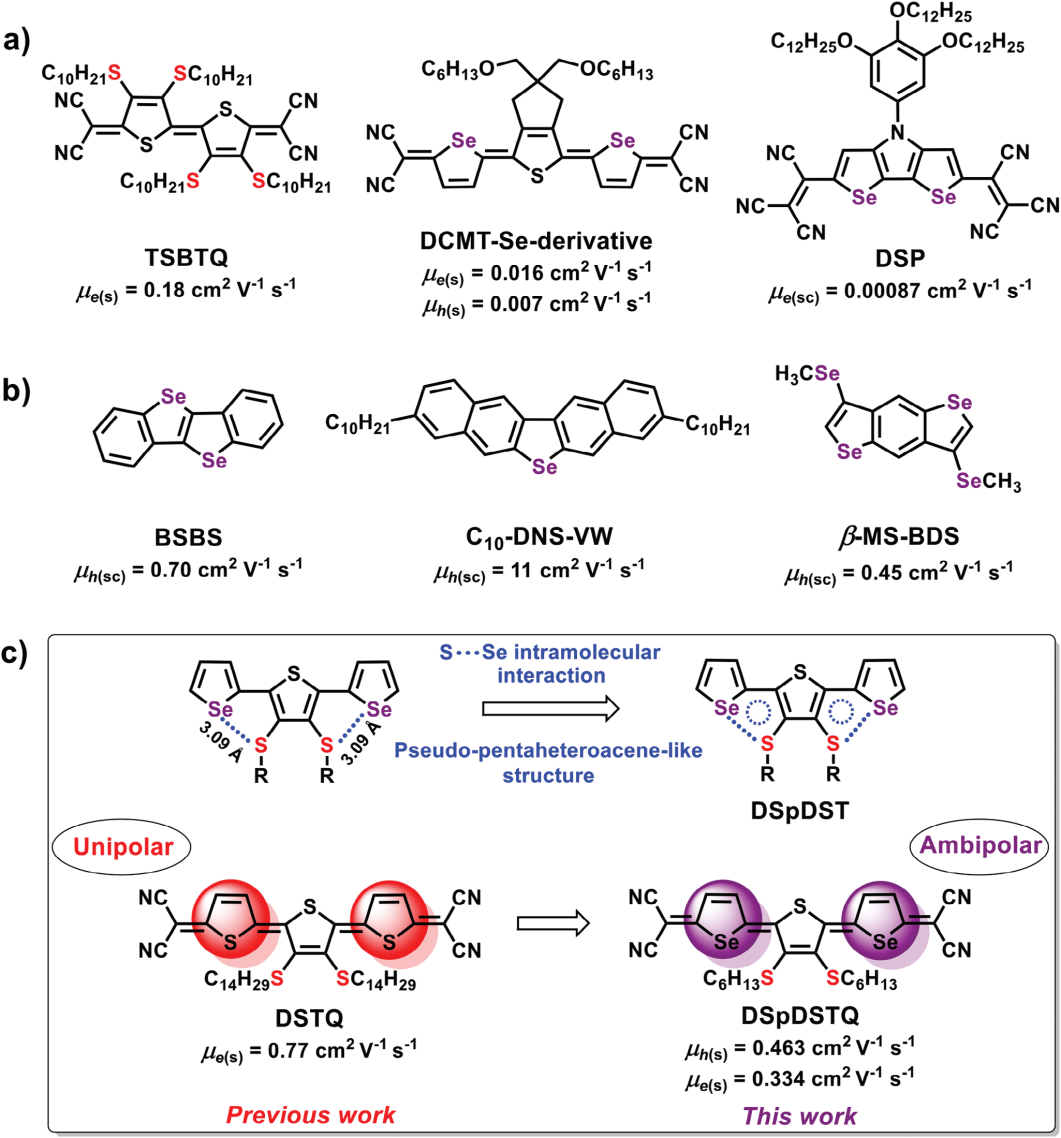
Chemical structures of the reported a) Thioalkyl/Se‐containing small molecules with dicyanomethylene end‐capping units, and b) Se‐containing fused small molecules for OFETs. c) Pseudo‐pentaheteroacene‐like structure of **DSpDST** core and the chemical structure of quinoids discussed in this study. *μ*_h_ and *μ*_e_ denote maximum hole and electron mobilities. The symbols (sc), and (s) denote spin coating and solution‐processed semiconductor films.

In view of the above considerations, various approaches have been explored for the development of high‐performance quinoidal oligothiophene‐based semiconductors, with a focus on achieving high planarity, close *π*–*π* stacking, and strong intermolecular or noncovalent interactions, in combination with heteroatom effects and alkyl chain modifications.^[^ ^36^, ^37^, ^38^, ^39^ ^]^ For example, the energy‐level modification of a thienoquinoidal semiconductor with electron‐withdrawing DCN‐end‐capping units, along with suitably solubilizing alkyl chains for increased film processability and the introduction of hetero‐atoms in the side chain to promote molecular orbital delocalization, has been shown to improve the device performance via strong intramolecular/conformational locking.^[^ ^34^, ^40^, ^41^, ^42^, ^43^, ^44^ ^]^ In addition, soluble dithioalkyl‐substituted organic semiconductors have been reported previously by the author's research group,^[^ ^35^, ^43^ ^]^ wherein the sulfur atoms of the thioalkyl chains provided intramolecular locks with the sulfur atoms of the thiophene (S···S), thereby enhancing the molecular planarity and charge transport efficiency.^[^ ^45^ ^]^ For example, thioalkyl side chains were successfully used to fabricate solution‐processable n‐type quinoidal organic semiconductors such as the tetra thioalkyl‐substituted TSBTQ in **Figure** **1**a, which exhibited an electron mobility of 0.18 cm^2^ V^−1^ s^−1^. Notably, the selection of a suitable thioalkyl side chain leads to the potential for an environmentally stable OFET.^[^ ^46^ ^]^

Selenium heterocycles, such as selenophene, possess distinct characteristics compared to their sulfur‐containing counterparts. Specifically, they are larger in size, more polarizable, and less electronegative.^[^ ^47^, ^48^ ^]^ In the solid state, selenium‐containing compounds demonstrate enhanced intermolecular interactions, including partial Se‐Se interactions, which are absent from their sulfur‐containing analogs. These Se‐Se interactions contribute to improved molecular ordering and unique solid‐state organization, often leading to excellent charge transport properties.^[^ ^49^, ^50^ ^]^ Thus, selenophene has been extensively utilized in the development of p‐type OFETs with outstanding performance.^[^ ^50^, ^51^, ^52^, ^53^, ^54^ ^]^ For example, by taking advantage of Se in the intramolecular lock series, the author's group has recently achieved significant advancements, with the selenotetradecyl‐substituted molecule DDTT‐SeBT exhibiting the highest hole mobility thus far reported for all/fused thiophene semiconductors (>4.0 cm^2^ V^−1^ s^−1^).^[^ ^55^ ^]^ Furthermore, chemical structures of the reported Se‐containing small molecules for OFETs have been given in Figure 1a,b. To investigate the extent of intermolecular overlap, selenophenes were introduced as replacements for thiophenes, and the resulting spin‐coated thin films of the quinoidal mixed oligomer DCMT‐Se‐derivative were shown to exhibit ambipolar carrier transport, with electron and hole mobilities of 0.016 and 0.007 cm^2^ V^−1^ s^−1^, respectively.^[^ ^56^ ^]^ Furthermore, Marder et al. reported the charge transport properties of DSP derivatives to achieve electron mobilities of up to 0.00087 cm^2^ V^−1^ s^−1^ in spin‐cast layers,^[^ ^57^ ^]^ while Takimiya et al. reported hole mobilities of up to 0.70 cm^2^ V^−1^ s^−1^ for single‐crystal OFETs based on the small molecule BSBS.^[^ ^58^ ^]^ Moreover, the latter research group also investigated *β*‐MS‐BDS, which is the only known methylseleno‐substituted small molecular semiconductor, and achieved a hole mobility of 0.45 cm^2^ V^−1^ s^−1^.^[^ ^59^ ^]^ Furthermore, Okamoto et al. reported an impressive charge‐carrier mobility of 11 cm^2^ V^−1^ s^−1^ for the alkyl‐substituted selenium‐bridged V‐shaped organic semiconductor C_10_‐DNS‐VW.^[^ ^60^ ^]^

The incorporation of selenophene into organic semiconductors has shown promising results in OFETs. Nevertheless, it is necessary to investigate the structure‐property relationship of the selenophene‐containing semiconducting materials.^[^ ^61^ ^]^ For example, the present authors have previously reported the synthesis of conjugated tetracyanoquinodimethane‐substituted quinoidal dithioalkylterthiophene (DSTQ) compounds such as DSTQ‐14 (Figure 1c), which features a dithiotetradecyl side chain and exhibits a superior electron mobility of 0.77 cm^2^ V^−1^ s^−1^.^[^ ^46^ ^]^ Hence, to further explore the structure‐property relationship and its influence on the OFET performance, the thiophene units of DSTQ‐14 compound are replaced with selenophene to obtain novel quinoidal compounds that incorporate distinct thioalkyl chains, namely **DSpDSTQ‐6** (1), with a dithiohexyl side chain (SR = SC_6_H_13_), **DSpDSTQ‐10** (2), with a dithiodecyl side chain (SR = SC_10_H_21_), and **DSpDSTQ‐14** (3), with a dithiotetradecyl side chain (SR = SC_14_H_29_). Further, to investigate the intramolecular lock property and intermolecular interactions, the optical, electrochemical, and thermal properties of the newly synthesized **DSpDSTQ**s are analyzed and compared. A crystal structure analysis of the **DSpDSTQ‐14** reveals a planar DSpDST core with highly ordered molecular packing, strong *π*–*π* interactions, intramolecular charge transfer between S(thioalkyl) and Se moieties, and intermolecular (N−H, and Se−Se) interactions among the face‐to‐face molecular layers. Bottom‐gate top‐contact (BGTC) OFET devices are then fabricated using both solution‐sheared and spin‐coated films to evaluate the charge transport characteristics. The results indicate that the **DSpDSTQ‐6**‐based solution‐sheared OFETs, featuring a dithiohexyl side chain, exhibit ambipolar charge‐carrier transport, along with the highest electron and hole mobilities of 0.334 and 0.463 cm^2^ V^−1^ s^−1^, respectively. This is attributed to the formation of aligned films with larger crystallites along the shearing direction and close molecular packing facilitated by noncovalent interactions, as revealed by the morphological and microstructural analyses. Furthermore, the air‐stable ambipolar characteristics of the **DSpDSTQ**‐based OFETs afford great potential for application as low‐cost, solution‐processable, ambipolar OFETs.

## 2 Results and Discussion

### 2.1 Synthesis

**Scheme 1:**
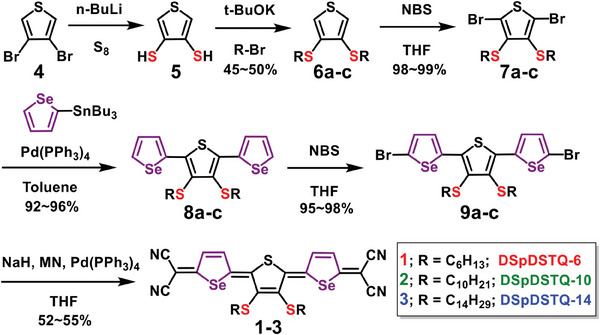
Synthetic route to quinoidal semiconductors **DSpDSTQ** (1‐3).

The synthetic route to quinoids 1−3 is shown in **Scheme** **1**. Compounds 4–7 were synthesized according to the previously reported procedure.^[^ ^46^, ^62^ ^]^ Briefly, the central dithioalkylated thiophene unit (6) was obtained by initial dilithiation of 3,4‐dibromothiophene (4) using n‐BuLi, followed by the reaction with sulfur and subsequent dialkylation with the appropriate alkyl bromide. Subsequent dibromination of 6 using n‐bromosuccinimide (NBS) provided the corresponding intermediate 7, which was then subjected to Stille coupling with 2‐(tributylstannyl)‐selenophene in the presence of a palladium catalyst to afford compound 8. Further treatment of 8 with NBS yielded the dibrominated intermediate 9. Finally, the desired quinoids **DSpDSTQ**(1−3) were obtained via Takahashi coupling of the compound 9 with malononitrile in the presence of tetrakis(triphenylphosphine)palladium, followed by oxidation using a saturated solution of bromine in water. The as‐synthesized quinoids exhibited good solubility in common organic solvents and were suitable for thin‐film solution processing. The chemical structures of all target compounds were confirmed by ^1^H NMR, ^13^C NMR, and mass spectrometry. Detailed information regarding the synthetic procedures and characterization can be found in the Supporting Information.

### 2.2 Physical Characterization

The thermal properties of the as‐synthesized **DSpDSTQ** (1–3) are revealed by the differential scanning calorimetry (DSC) and thermogravimetric analysis (TGA) results in Figure S1 and S2 (Supporting Information), respectively, and are summarized in **Table** **1**. The DSC scans clearly indicate distinct phase transitions for each of the compounds, characterized by sharp endothermic peaks above 196 °C. Notably, the melting points of the **DSpDSTQ** molecules are seen to decrease with the decreasing length of the alkyl side chain, i.e.: 1 (224 °C) > 2 (209 °C) > 3 (196 °C). This trend is also reflected in the melting temperatures, i.e.: 1 (229 °C) > 2 (214 °C) > 3 (209 °C). Further, the TGA analysis demonstrates that all three **DSpDSTQ** molecules exhibit high thermal stability, with an initial weight loss of ≈ 5% occurring in the temperature range of 291–307 °C.

**Table 1:** Thermal, Optical, and Electrochemical properties of all three Compounds.

Compound	*T*_d_ ^a)^ [°C]	*T*_m_ ^b)^ [°C]	*λ*_max_(sol)^c)^ [nm]	*λ*_max_(film)^d)^ [nm]	*E*_ox_ ^e)^ [V]	HOMO^f)^ [eV]	*E*_red_ ^e)^ [V]	LUMO^f)^ [eV]	Δ*E*_g_^g^ [eV]
**DSpDSTQ‐6** (1)	307	224	707	713	1.40	−5.60	0.02	−4.22	1.38
**DSpDSTQ‐10** (2)	291	209	708	713	1.40	−5.60	0.02	−4.22	1.38
**DSpDSTQ‐14** (3)	295	196	707	726	1.40	−5.60	0.01	−4.21	1.39

^a)^ Decomposition temperatures were determined from TGA;

^b)^ Melting temperatures were determined from DSC;

^c)^ Absorption spectra were measured in *o*‐C_6_H_4_Cl_2_;

^d)^ Thin films were solution‐sheared onto a quartz glass;

^e)^ By DPV in *o*‐C_6_H_4_Cl_2_ at 25 °C. All potentials are reported with reference to an Fc/Fc^+^ internal standard (at +0.6 V);

^f)^ Using HOMO/LUMO = −(4.2 + *E*_ox_/*E*_red_);

^g)^ The energy gap was calculated from the difference between HOMO and LUMO measured by DPV.

**Figure 2:**
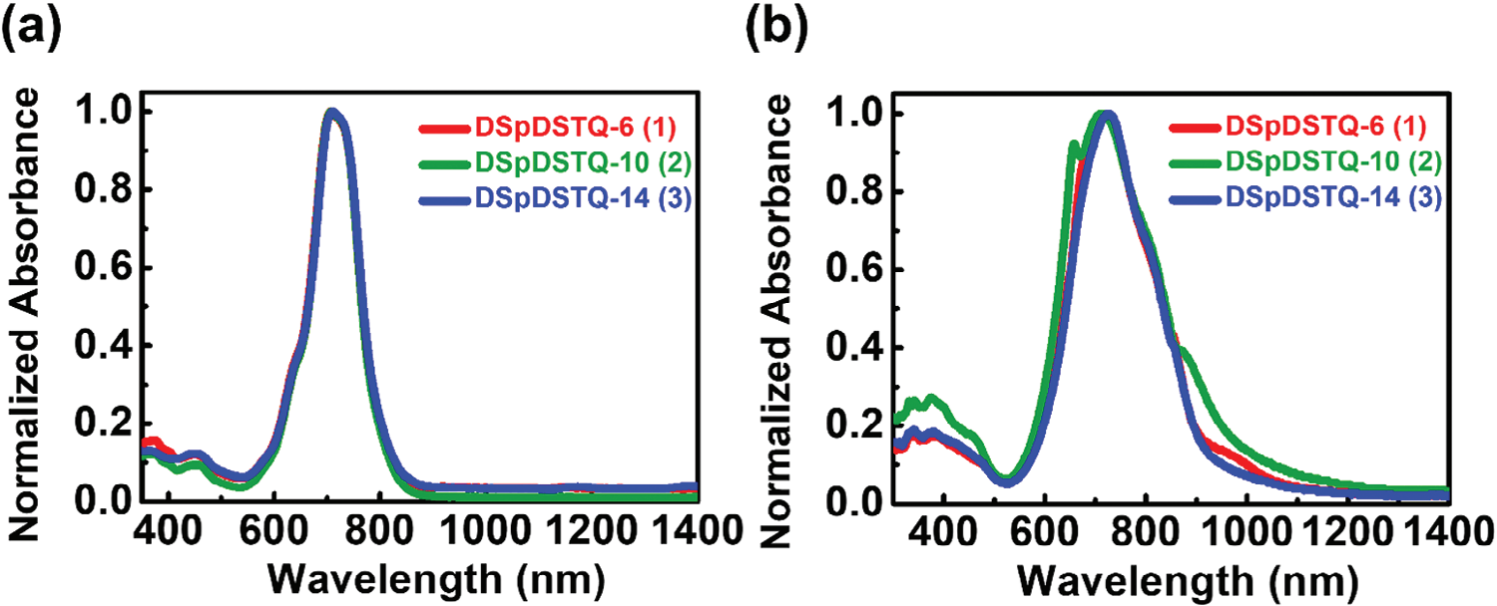
UV–vis absorption spectra of **DSpDSTQ**s in a) dilute *o*‐dichlorobenzene and b) solution‐sheared film state.

The UV‐vis absorption spectra of the **DSpDSTQ** compounds in diluted o‐dichlorobenzene solution and solution‐sheared films are presented in **Figure** **2**. Here, the solution‐state spectra are seen to be very similar, with a maximum absorption wavelength (*λ_max_*) centered at ≈707 nm (Figure 2a). This suggests that the thioalkyl side chain substituent does not exert a significant influence on the *π*‐conjugated quinoidal backbone of each compound, and is consistent with previous studies.^[^ ^63^, ^64^ ^]^ Notably, all three solution‐sheared films exhibit substantial bathochromic shifts compared to the solution phase with Δ*λ* values of 6, 5, and 19 nm for **DSpDSTQ‐6**, **DSpDSTQ‐10**, **DSpDSTQ‐14**, respectively. These shifts can be attributed to both the different extent of aggregation behavior and distinct electronic coupling and packing modes of the **DSpDSTQ** molecules in solution‐sheared films.^[^ ^65^, ^66^ ^]^ The intense, low‐energy absorption bands in the range of 600–800 nm are assigned to *π*–π* transitions with intramolecular charge transfer character between the electron‐rich and electron‐deficient moieties, while the weaker high‐energy bands in the range of 350–450 nm are ascribed to localized electronic transitions of the quinoidal backbone.^[^ ^67^ ^]^ The broader absorption profiles of the solution‐sheared **DSpDSTQ** films relative to those obtained in the solution state can also be explained by molecular aggregation.^[^ ^68^ ^]^

**Figure 3:**
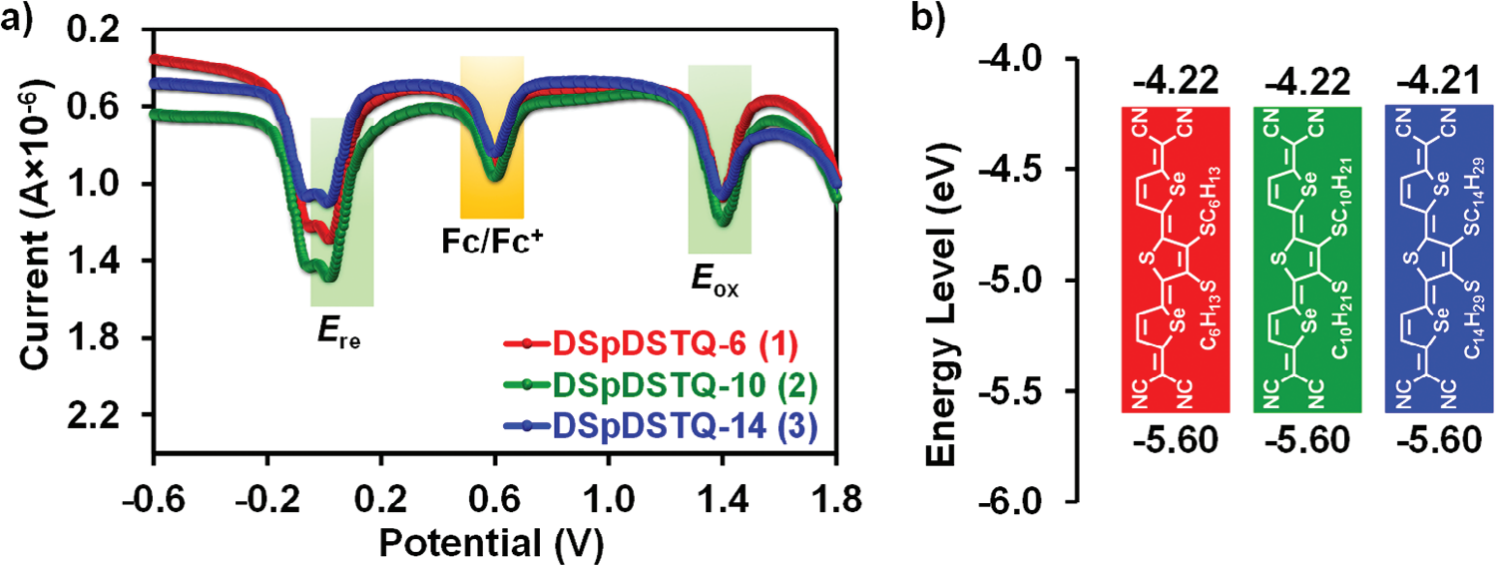
a) DPV of **DSpDSTQ**s in *o*‐dichlorobenzene. b) DPV‐derived HOMO and LUMO energy levels. All potentials reported are referenced to an Fc/Fc^+^ internal standard (at +0.6 V).

The electrochemical properties of the three **DSpDSTQ**s are revealed by the differential pulse voltammetry (DPV) results in **Figure** **3** and **Table** 1. Here, all of the quinoidal molecules exhibit similar oxidation and reduction potentials, thereby indicating that the alkyl substituent has little effect on the electrochemical properties of the **DSpDSTQ**. Consequently, by using the equation HOMO/LUMO = −(4.2 + *E*_ox_/*E*_red_), and assuming an internal standard ferrocene/ferrocenium (Fc/Fc^+^) oxidation at −4.8 eV, the LUMO and HOMO values of all three compounds are calculated to be ≈ −4.2 and −5.6 eV, respectively. Moreover, the electrochemically derived HOMO‐LUMO energy gap (*E*_g_) of each compound is as low as ≈1.38 eV, thereby demonstrating that the **DSpDSTQ**s are favorable as a new class of semiconducting materials for use in OFETs. Furthermore, all three **DSpDSTQ**s exhibit a LUMO energy of ≈ −4.2 eV, thereby indicating their suitability for efficient charge transfer and the development of air‐stable, solution‐processable OFETs.

### 2.3 Theoretical Calculations

The frontier molecular orbitals of the **DSpDSTQ** compounds are elucidated by the results of electronic structure calculations at the B3LYP/6‐311(d,p) level of density functional theory (DFT) in Figure S3 (Supporting Information). Despite the varying lengths of the thioalkyl chains, all of the calculated compounds exhibit similar electron density distributions, with HOMO and LUMO being extensively delocalized over the entire quinoidal backbone and sulfur atoms. This significant delocalization of the quinoidal structure may partly explain the observed ambipolar behavior. Moreover, ambipolar materials typically possess a small bandgap that matches the required HOMO/LUMO levels for efficient injection of electrons and holes from a single electrode material.^[^ ^69^, ^70^ ^]^ All three **DSpDSTQ** derivatives meet the requirement with a low bandgap of 1.56 eV. The exceptional LUMO level of –4.44 eV stems from the stronger electron‐withdrawing nature of the cyano group, which provides better resistance to water and oxygen, making it a potential candidate for environmentally stable OFETs.^[^ ^71^, ^72^, ^73^, ^74^ ^]^ Further, the DFT calculations for the **DSpDSTQ** core indicate high planarity, with a skeletal distortion of <8° (Figure S3a, Supporting Information), which is beneficial for charge transport by promoting efficient delocalization of the *π*−electrons and facilitating *π*−*π* overlap and intermolecular interactions. This result also confirms the presence of the intramolecular locks provided by the S···Se interactions,^[^ ^43^ ^]^ with a shorter Se(core)··S(R) distance of 3.08 Å, and a larger selenophene distortion angle of 7.31°, compared to 3.12 Å and 2.89° for the previously‐reported S···S interaction and thiophene distortion, respectively.^[^ ^46^ ^]^ This result can be attributed to the greater steric bulk of the selenium atom, which also leads to larger polarizability, thereby enhancing the charge transfer.^[^ ^75^, ^76^ ^]^ The energy difference between the aromatic and quinoid forms (Δ*E* = *E*_quinoid_− *E*_aromatic_, kcal mol^−1^) for selenophene is relatively smaller than for thiophene.^[^ ^77^ ^]^ This indicates that selenophenes have a more quinoidal character than their thiophene counterparts, which increases the efficiency of conjugation through the backbone and lowers the reorganization energy.^[^ ^36^ ^]^ Additionally, this effect promotes the destabilization of the ground state of the molecule toward a quinoidal configuration, which effectively lowers the band gap in the conjugated system.^[^ ^78^ ^]^

### 2.4 Single Crystal Structure

**Figure 4:**
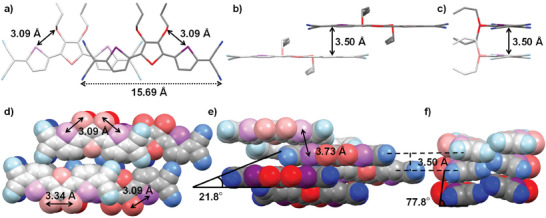
Single‐crystal structure of **DSpDSTQ‐14** (3) in stick models (a−c) and space‐filling packing models (d−f). a) Top view of 3 with intramolecular S(thioalkyl)−Se distance of 3.09 Å and the molecular length of 15.69 Å. b) Front view of two stacking **DSpDSTQ** molecules. The interplanar distance is ∼3.50 Å. c) Side view of 3. d) Short intramolecular S(thioalkyl)−S(thioalkyl) distance of 3.34 Å and S(thioalkyl)−Se is 3.09 Å. (e−f) Molecular packing arrangement of **DSpDSTQ** molecules with a face‐to‐face layer stacking distance of 3.50 Å and exhibiting the slipping angles of 21.8° and 77.8°. The shortest intermolecular Se−Se distance of 3.73 Å. The selenium, sulfur, and nitrogen atoms are specified in purple, red, and blue color, respectively. The alkyl chains are omitted for clarity.

As a representative example, the diffraction‐derived single crystal structure of the **DSpDSTQ‐14** is presented in **Figure** **4** and Figure S4 (Supporting Information), and summarized in Table S1 (Supporting Information). These results indicate that the **DSpDSTQ‐14** crystallizes in the triclinic space group *P*1 and adopts a face‐to‐face slipped *π‐*‐*π* stacking arrangement with a stacking distance of 3.50 Å. This stacking configuration is believed to be highly conducive to efficient charge transport.^[^ ^40^, ^46^ ^]^ Within the crystal structure, the shortest intramolecular distance between S(thioalkyl) and Se(selenophene) is measured as 3.09 Å, while the shortest distance between S(thioalkyl) and S(thioalkyl) is found to be 3.34 Å (Figure 4a,d). The front and side views of two stacked **DSpDSTQ** molecules with a short packing distance of 3.50 Å are depicted in Figure 4b,c. The shortest intermolecular N···H and Se···Se distances among the face‐to‐face layers of the **DSpDSTQ‐14** molecules are found to be 2.71−2.72 Å (Figure S4b,c, Supporting Information) and 3.73 Å (Figure 4e) respectively. These distances are significantly smaller than the sum of the Van der Waals radii of the respective atoms, thereby indicating the presence of strong intra‐ and intermolecular interactions. The molecular packing arrangement of the **DSpDSTQ** molecules exhibits slipping angles of 21.8° and 77.8° along the long and short molecular axes, respectively (Figure 4e,f). Based on these results, it can be concluded that the **DSpDSTQ‐14** quinoids possess planar molecular cores with short packing distances, thereby suggesting favorable intra‐ and intermolecular non‐bonded contacts. These characteristics are expected to minimize the occurrence of morphological defects and enhance charge transport in the OFETs.

### 2.5 Charge Transport Measurements

**Figure 5:**
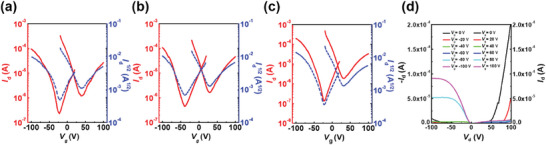
Representative transfer characteristics of solution‐sheared OFETs based on a) **DSpDSTQ‐6**, b) **DSpDSTQ‐10**, c) **DSpDSTQ‐14** thin films (under a constant *V*_d_ of 100 V for n‐type and –100 V for p‐type measurements); d) Output characteristics of solution‐sheared OFETs based on **DSpDSTQ‐6** thin films. All the OFETs were measured in the glove box at room temperature.

To obtain a comprehensive understanding of the impact of the semiconducting properties of **DSpDSTQ** compounds with different lengths of thioalkyl chains, bottom‐gate top‐contact (BGTC) OFETs were fabricated using both solution‐shearing and spin‐coating methods for comparison, as detailed in the Supporting Information. The solution‐shearing method allows for the aligned growth of semiconductor crystals by controlling various processing parameters such as shearing speed, substrate temperature, solvent choice, and semiconductor concentration.^[^ ^79^, ^80^, ^81^, ^82^ ^]^ This method enables the formation of an optimized film with well‐aligned structures under the influence of the shearing force. It is worth noting that **DSpDSTQ** derivatives exhibit great solubility in ortho‐dichlorobenzene (*o*‐DCB), making them suitable for solution processing. Detailed information on OFET device fabrication and measurement procedures can be found in the Experimental Section. Representative transfer and output curves of the as‐fabricated OFETs are presented in **Figure** **5**. Here, the transfer characteristics revealed by the drain current‐gate voltage (*I*_d_−*V*_g_) curves clearly indicate the ambipolar behavior of all three **DSpDSTQ** compounds (Figure 5a–c). This indicates that the substitution of the larger and more polarizable Se atom has a positive influence on efficient carrier injection from gold electrodes, resulting in balanced ambipolar mobilities at room temperature.^[^ ^83^ ^]^ Moreover, the drain current‐drain voltage (*I*_d_−*V*_d_) curve of the **DSpDSTQ‐6** compound reveals a significant increase in the channel output current at low *V*_g_ and high *V*_d_ values (Figure 5d). This behavior is a characteristic of ambipolar charge transport,^[^ ^84^, ^85^, ^86^ ^]^ indicating the ability of this compound to support both electron and hole transport.

The threshold voltage (*V*_th_) is extracted from the slope and intercept of $\sqrt{I_{d}}$ versus *V*_g_ in the saturation regime, and the charge carrier mobilities (μ) are calculated according to gradual‐channel approximation in the saturation region by the equation below:

(1) $$\mu =\frac{2L}{CW}{\left(\frac{\partial \sqrt{I_{d}}}{\partial V_{g}}\right)}^{2}$$

where *C* is the capacitance per unit area of the dielectric layer, L is the channel length (25 µm), and W is the channel width (1500 µm). The device parameters, including maximum mobility (*μ*_max_), average mobility (*μ*_avg_), *V*_th_, and current ON/OFF ratios (I_ON_/I_OFF_) are summarized in **Table** **2**. Notably, the solution‐shearing devices exhibit clear thioalkyl side chain‐dependent and electrical anisotropy. Thus, as the side chain length increases, the mobility decreases, with the **DSpDSTQ‐6**, **DSpDSTQ‐10**, and **DSpDSTQ‐14** exhibiting electron μ_avg_ values of 0.182 ± 0.083, 0.101 ± 0.046, and 0.079 ± 0.042 cm^2^ V^−1^ s^−1^, respectively, and hole *μ*_avg_ values of 0.203 ± 0.098, 0.095 ± 0.028 and 0.065 ± 0.023 cm^2^ V^−1^ s^−1^, respectively. Hence, the **DSpDSTQ‐6** exhibits the highest electron and hole *μ*_max_ values of 0.334 and 0.463 cm^2^ V^−1^ s^−1^, respectively. Additionally, it is observed that the parallel direction shows a consistently higher *μ* compared to the perpendicular direction, indicating a preferable charge transport by shearing‐induced crystal growth.

**Table 2:** Summary of OFET Parameters Based on Solution‐Sheared **DSpDSTQ** Thin Films, as Measured in the glove box at Room Temperature.

Compound	Shearing Direction	n‐channel			p‐channel		
		*μ*_avg_ ^a)^ (*μ*_max_ ^b)^) [cm^2^ V^−1^ s^−1^]	*V*_th_ [V]	*I*_ON_/*I*_OFF_ [‐]	*μ*_avg_ ^a)^ (*μ*_max_ ^b)^) [cm^2^ V^−1^ s^−1^]	*V*_th_ [V]	*I*_ON_/*I*_OFF_ [‐]
**DSpDSTQ‐6** (1)	parallel	0.182 ± 0.083 (0.334)	28.2 ± 4.2	10^2^–10^3^	0.203 ± 0.098 (0.463)	−39.0 ± 10.5	10^2^–10^4^
	vertical	0.153 ± 0.052 (0.241)	42.1 ± 10.9	10^2^–10^3^	0.197 ± 0.133 (0.343)	−55.5 ± 9.3	10^2^
**DSpDSTQ‐10** (2)	parallel	0.101 ± 0.046 (0.163)	27.6 ± 2.9	10^2^–10^3^	0.095 ± 0.028 (0.144)	−44.1 ± 13.3	10^2^–10^3^
	vertical	0.067 ± 0.042 (0.137)	28.2 ± 1.7	10^2^–10^3^	0.045 ± 0.010 (0.061)	−28.9 ± 12.7	10^2^–10^3^
**DSpDSTQ‐14** (3)	parallel	0.079 ± 0.042 (0.156)	35.3 ± 10.2	10^2^–10^3^	0.065 ± 0.023 (0.100)	−15.5 ± 5.6	10^2^–10^4^
	vertical	0.044 ± 0.019 (0.072)	33.4 ± 6.7	10^2^–10^3^	0.054 ± 0.017 (0.085)	−23.4 ± 10.5	10^2^–10^5^

^a)^ The average TFT characteristics were obtained from >10 devices originating from 3 to 4 semiconductor depositions;

^b)^ Maximum mobility.

The relatively low ON/OFF ratio can be attributed to the thicker semiconductor film (70–120 nm) produced by solution‐shearing, which specifically leads to a higher OFF current causing the unideal ON/OFF ratio. Meanwhile, the large *V_th_* and switch‐on voltage indicate the existence of deep trap states at the interface between the semiconductor film and the dielectric.^[^ ^87^, ^88^ ^]^ Mobile carriers may interact with shallow trap states in the vicinity of a chemical impurity, allowing the capture of the electron into a deeply trapped state.^[^ ^89^, ^90^ ^]^ Consequently, the present results are comparable with those of previous studies, where transistors based on the majority of organic semiconductor crystals with ambipolar characteristics by using air‐stable electrode materials such as gold, would have required relatively high operating voltages for the simultaneous injection and transport of the two opposite carriers. The fact can be attributed to high ohmic contact in the crystal/metal interface, which indicates the existence of an injection barrier.^[^ ^91^, ^92^, ^93^ ^]^

For comparison, representative transfer curves of the spin‐coated **DSpDSTQ‐6**‐based devices are presented in Figure S5 (Supporting Information). There, typical ambipolar characteristics are again observed. However, the manifestation of p‐type behavior occurred at high *V_g_*, where the hole mobility cannot be calculated accurately under this circumstance. The extracted electron mobility of the spin‐coated **DSpDSTQ‐6** OFETs is 9.84 × 10^−3^ cm^2^ V^−1^ s^−1^, which is over one order of magnitude lower than the solution‐sheared counterparts.

### 2.6 Thin Film Morphological and Microstructural Analysis

**Figure 6:**
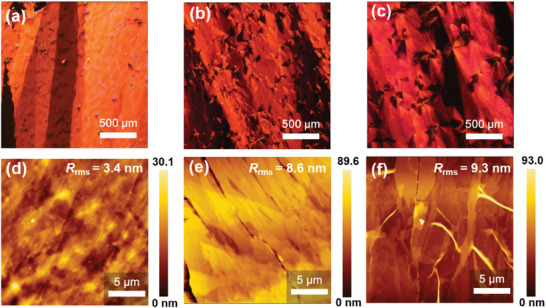
POM images of solution‐sheared a) **DSpDSTQ‐6**, b) **DSpDSTQ‐10**, and c) **DSpDSTQ‐14**; AFM images of solution‐sheared d) **DSpDSTQ‐6**, e) **DSpDSTQ‐10**, and f) **DSpDSTQ‐14**.

To understand the effects of side‐chain engineering on the OFET performance of the **DSpDSTQ** thin films, the morphologies are examined via polarized optical microscopy (POM; **Figure** **6**a‐c) and by atomic force microscopy (AFM; Figure 6d‐f). Herein, the POM images reveal large crystalline domains with millimeter‐scale dimensions along the shearing direction. Further, **DSpDSTQ‐10** and **DSpDSTQ‐14** films exhibit randomly distributed spherulitic precipitates with a cross structure. This phenomenon is driven by strong solvent evaporation at the nucleation sites, which generates a concentration gradient and induces supersaturation, thereby initiating the crystallization process near the aggregation points.^[^ ^94^ ^]^ Meanwhile, AFM topographic images present that **DSpDSTQ‐6** contains well‐interconnected crystals with a terrace‐like morphology, while both **DSpDSTQ‐10** and **DSpDSTQ‐14** show more cracks and grain boundaries. The presence of grain boundaries can create an energy barrier and limit the charge carrier mobility in organic semiconductor films. Moreover, the root mean square roughness (*R*_rms_) of the thin film increases from 3.4 nm for the **DSpDSTQ‐6** to 8.6 and 9.3 nm for the **DSpDSTQ‐10** and **DSpDSTQ‐14**, respectively, due to the increase in side‐chain length. The smoother surface of **DSpDSTQ‐6** allows for more efficient charge transport with fewer barriers and grain boundaries, which is consistent with its better electrical performance reported in **Table** 2. By contrast, the longer insulating alkyl chains of the **DSpDSTQ‐10** and **DSpDSTQ‐14** may hinder the charge transport efficiency, thereby causing a lower field effect mobility. For comparison, the OM and POM images of the spin‐coated **DSpDSTQ‐6** OFETs are presented in Figure S6 (Supporting Information), where the fast and random growth of crystals has resulted in a discontinuous and non‐oriented crystalline morphology. Overall, the above thin film morphological analysis reveals the influence of side chain length on the crystal structure, surface roughness, and interconnectivity of the **DSpDSTQ** films, which ultimately impacts the charge transport properties in the OFETs.

**Figure 7:**
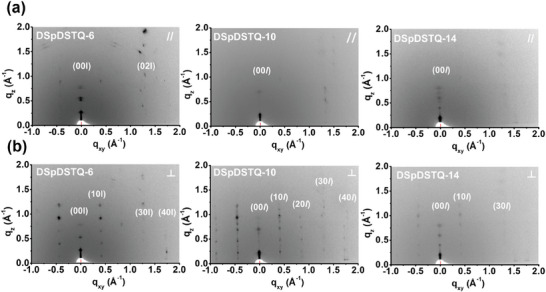
2D GIXRD patterns of solution‐sheared **DSpDSTQ‐6**, **DSpDSTQ‐10**, and **DSpDSTQ‐14** films with an incident beam along the a) parallel (//) and b) perpendicular (⊥) directions with respect to the shearing direction.

In addition, the molecular packing structures of the **DSpDSTQ** compounds are revealed by the 2D grazing‐incidence wide‐angle X‐ray scattering (GIXRD) patterns obtained with the incident beam parallel and perpendicular to the shearing direction in **Figure** **7**. The unit cell parameters are defined in Figure S7 (Supporting Information), where the *a*, *b*, and *c* axes represent the *π–π* interaction distance, the main core length, and the length of the alkyl side chain, respectively. The various parameters were determined from the change in incident beam direction, as shown in Figure S8 (Supporting Information), where the in‐plane (*q*_xy_) direction represents the *π–π* interaction distance parallel to the shearing direction and the main core length perpendicular to the shearing direction, while the out‐of‐plane direction (*q*_z_) reflects the alkyl side‐chain length regardless of the incident beam direction. The (00*l*) lamellar diffractions in the out‐of‐plane (*q*_z_) direction were clearly detected for all films, indicating a dominant edge‐on molecular orientation with the side chains aligned perpendicular to the substrate. The lamellar distance (*d*_001_) values are determined from the primary peaks as 23.6, 26.6, and 29.7 Å for the **DSpDSTQ‐6**, **DSpDSTQ‐10**, and **DSpDSTQ‐14**, respectively. This is consistent with the increase in alkyl chain length (Table S2, Supporting Information).

A comparison between the GIXRD patterns in Figure 7a,b indicates that all of the **DSpDSTQ**s exhibit more and stronger diffraction peaks in the perpendicular direction than in the parallel direction. This indicates a greater degree of orientation in the microstructure induced by solution‐shearing method. Among all the compounds, **DSpDSTQ‐6** exhibits the sharpest diffraction peaks in both parallel and perpendicular directions, indicating the most ordered layered structure and highest crystallinity. Furthermore, only **DSpDSTQ‐6** film shows the (02*l*) diffractions in the parallel direction, representing the periodic structure along the conjugated backbone in *b*‐axis. Meanwhile, the (10*l)*, (20*l*), (30*l*), and (40*l*) diffraction peaks in perpendicular direction correspond to the 𝜋‐𝜋 stacking of the conjugated backbones along *a*‐axis, which provides a more efficient pathway for charge carriers to transport. This is the reason why the **DSpDSTQ** films display higher mobilities along the parallel direction than the perpendicular direction. In the case of the spin‐coated films (Figure S9, Supporting Information), only the **DSpDSTQ‐6** exhibits a clear (001) diffraction peak, while the other films show amorphous GIXRD patterns. This confirms that the solution‐shearing method directly affects the anisotropic behavior of the **DSpDSTQ** film microstructure.

Based on the abovementioned GIXRD diffraction patterns and single‐crystal data, the molecular packing behavior in a thin‐film state can be proposed. A comparison of the length of the *c*‐axis from single‐crystal data and lamellar distance from GIXRD indicates that the **DSpDSTQ** molecules are aligned with the *c*‐axis, as shown in Figure S8 (Supporting Information). The AFM height profile in Figure S10 (Supporting Information) provides some information on the surface crystallographic plane of extended growth terraces. In comparison with the DFT calculation, the GIXRD results and height profile data demonstrate a longer side chain length. This can be attributed to the solution‐shearing process, inducing lattice distortion and elongating the c‐axis. Moreover, the step height of the **DSpDSTQ‐6** (2.4 nm) is consistent with the GIXRD‐derived single layer (2.38 nm), thus suggesting the formation of a monolayer on the terrace structure.

The coherence length (*L_c_*), which provides information about the grain size, can be determined from the GIXRD data. Specifically, after determining the full width at half maximum (FWHM) by integrating the entire primary peak, the *L_c_* value is given by the Scherrer equation, Equation (2):

(2) $$L_{c}=\frac{K\lambda }{\beta \cos\theta }$$

where K is the constant of integrated FWHM (K = 0.89), λ is the wavelength of XRD radiation, β is the FWHM in radians, θ is the Bragg diffraction angle.^[^ ^95^ ^]^ The FWHM values were found to be 0.18, 0.23, and 0.32 Å^−1^ for **DSpDSTQ‐6**, **DSpDSTQ‐10**, and **DSpDSTQ‐14** films, respectively, resulting in *L_c_* values of 296.0 Å, 248.0 Å, and 178.0 Å. Larger grain sizes indicate fewer grain boundaries and better charge transport. Therefore, the inferior charge transport and lowest mobility of the **DSpDSTQ‐14** film can be explained by its smallest grain size and the presence of cracks in the crystals, as imaged by AFM. Conversely, the **DSpDSTQ‐6** provides the highest OFET mobility because it has the largest *L_c_* value, along with a highly ordered, highly crystalline structure, as indicated by its strong diffraction.

### 2.7 Device Stability Measurement

**Figure 8:**
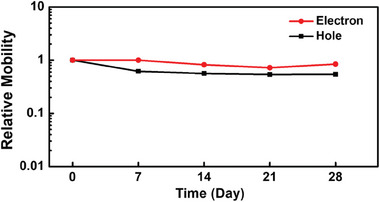
Ambient stability of **DSpDSTQ‐6** OFETs stored at a relative humidity of 35–45% and room temperature.

Finally, the environmental stabilities of the OFETs are evaluated by examining their performances under ambient conditions over an extended period. In the **DSpDSTQ** compounds, the introduction of strong electron‐withdrawing groups such as cyano groups, results in a deep‐lying LUMO level below –4.0 eV. This modification helps minimize water and oxygen‐induced trapping and improves the intrinsic environmental stability of the organic semiconductors. In the stability test, non‐encapsulated/non‐passivated **DSpDSTQ‐6** OFETs with top gold electrodes were monitored for hole and electron mobility over a period of 28 days under ambient conditions (35‐45% relative humidity and room temperature). The results in **Figure** **8** demonstrate that all of the devices exhibit good performance throughout the test duration, particularly in terms of stable electron transport. This indicates that the **DSpDSTQ‐6** provides good air stability, making it a promising candidate for application in low‐cost and solution‐processable ambipolar OFETs with the capacity for long‐term device operation without significant performance degradation. In brief, by tailoring the chemical structure and optimizing the LUMO level, the organic semiconductor material can be provided with greater stability, which is a crucial factor for practical OFET applications.

Developing ambipolar materials that can effectively inject both electrons and holes from a single electrode material is crucial for streamlining circuit design in real‐world applications. Common organic semiconductors often exhibit unipolar behavior and have rather broad band gaps, typically between 2–3 eV. One efficient way to reduce bandgap is to construct donor‐acceptor moieties, in which strong electron acceptors are co‐polymerized with electron‐rich donor monomers. In order to facilitate electron transport, a low LUMO molecule design strategy has been employed because the work function of common electrode materials is more aligned to HOMO than LUMO. Since the hybridization of the molecular orbitals, LUMO is dominated by the electron‐accepting unit. This work is dedicated to molecular design for **DSpDSTQ**, adding strong electron‐withdrawing functional groups such as the cyano group can greatly lower LUMO level and boost air stability simultaneously.

## 3 Conclusion

A novel series of small‐molecule quinoidal semiconductor materials based on diselenophene‐dithioalkylthiophene (designated **DSpDSTQ**) were developed herein for application in organic field‐effect transistors (OFETs). By varying the thioalkyl side chain length, compounds with distinct morphologies and microstructures were obtained, thereby modulating the electrical performance. In particular, the compound with the shortest side chain, designated **DSpDSTQ‐6**, exhibited the highest maximum electron and hole mobilities of 0.334 and 0.463 cm^2^ V^−1^ s^−1^, respectively, as evidenced by optical absorption, electronic/crystal structure, surface morphology, and solid‐state molecular packing analyses. The superior performance of the **DSpDSTQ‐6** was ascribed to the formation of aligned films with larger crystallites along the shearing direction, as evidenced by charge transport anisotropy measurements. Furthermore, the number of grain boundaries was reduced due to the presence of well‐connected semiconductor domains, thereby facilitating efficient charge transport, as confirmed by the largest coherence length and closely packed molecular stacking. Overall, the **DSpDSTQ**‐based OFETs exhibited excellent ambipolar characteristics and stable charge transport, making them promising solution‐processable and air‐stable organic semiconductor candidates for various OFET applications. This research not only advances the field of organic electronics but also underscores the potential of **DSpDSTQ**‐based compounds as valuable building blocks in the design and development of high‐performance organic electronic devices.

## 4 Experimental Section

### General Procedures for Final Target Compounds (1–3)

Malononitrile (4 equiv.) was added into a solution of sodium hydride (10 equiv.) in dry THF (20 mL) at 0 °C, and the mixture was warmed to room temperature and stirred for 30 min. 5,5′‐(3,4‐bis(alkylthio)thiophene‐2,5‐diyl)bis(2‐bromoselenophene) (9) (1 equiv.) and tetrakis(triphenylsphosphine)palladium (0.2 equiv.) were then added. The mixture was refluxed for 12 h, and then saturated bromine water was added slowly at 0 °C. The mixture was extracted with CH_2_Cl_2_, washed with brine, dried over Na_2_SO_4_, and evaporated. The residue was purified by column chromatography using dichloromethane/hexane followed by recrystallization from toluene yielding the desired quinoidal compound was obtained as a blue‐green solid.

### Synthesis of **DSpDSTQ‐6** (1)

^1^H NMR (500 MHz, CDCl_3_): δ (ppm) 7.66 (d, *J =* 5.9 Hz, 2H), 7.44 (d, *J =* 5.9 Hz, 2H), 3.05 (t, *J =* 7.5 Hz, 4H), 1.71‐1.65 (m, 4H), 1.46‐1.40 (m, 4H), 1.33‐1.25 (m, 8H), 0.9‐0.87 (m, 6H). ^13^C NMR (125 MHz, CDCl_3_): δ (ppm) 176.47, 150.94, 143.42, 142.35, 134.94, 133.25, 114.67, 113.15, 73.71, 38.66, 31.20, 29.63, 28.48, 22.48, 13.97. HRMS (MALDI, [M]^+^) calcd. for C_30_H_30_N_4_S_3_Se_2:_ 701.9963, Found: 701.9958.

### Synthesis of **DSpDSTQ‐10** (2)

^1^H NMR (500 MHz, CDCl_3_): δ (ppm) 7.66 (d, *J =* 6.0 Hz, 2H), 7.44 (d, *J =* 6.0 Hz, 2H), 3.05 (t, *J =* 7.4 Hz, 4H), 1.71‐1.65 (m, 4H), 1.45‐1.39 (m, 4H), 1.29‐1.25 (m, 24H), 0.89‐0.86 (m, 6H). ^13^C NMR (125 MHz, CDCl_3_): δ (ppm) 176.46, 150.91, 143.40, 142.32, 134.93, 133.24, 114.67, 113.13, 73.73, 38.61, 31.87, 29.66, 29.50, 29.47, 29.27, 29.03, 28.80, 22.67, 14.10. HRMS (MALDI, [M]^+^) calcd. for C_38_H_46_N_4_S_3_Se_2:_ 814.1215, Found: 814.1210.

### Synthesis of **DSpDSTQ‐14** (3)

^1^H NMR (500 MHz, CDCl_3_): δ (ppm) 7.66 (d, *J =* 5.9 Hz, 2H), 7.45 (d, *J =* 6.0 Hz, 2H), 3.05 (t, *J =* 7.5 Hz, 4H), 1.71‐1.65 (m, 4H), 1.45‐1.39 (m, 4H), 1.31‐1.25 (m, 40H), 0.89‐0.86 (m, 6H). ^13^C NMR (125 MHz, CDCl_3_): δ (ppm) 176.45, 150.91, 143.37, 142.30, 134.92, 133.25, 114.67, 113.12, 73.76, 38.61, 31.92, 29.67, 29.56, 29.47, 29.35, 29.05, 28.81, 22.68, 14.10. HRMS (MALDI, [M]^+^) calcd. for C_46_H_62_N_4_S_3_Se_2:_ 926.2467, Found: 926.2462.

### Device Fabrication and Measurement

The fabrication of the OFET devices followed a standard bottom‐gate top‐contact (BGTC) structure. Highly n‐doped silicon wafers with a thickness of 300 nm of thermally grown SiO_2_ were subjected to sonication for 5 min each in acetone and isopropanol. The wafers were then dried with nitrogen gas and treated with UV ozone plasma for 5 min to ensure surface cleanliness. To passivate the dielectric surface, a self‐assembly monolayer (SAM) was formed using (2‐phenylethyl) trichlorosilane (PETS) from a 1 mM solution in toluene. The silicon substrates were immersed in the PETS solution at a temperature of 55 °C for 1 h, allowing the SAM formation. The **DSpDSTQ** compounds were dissolved in o‐chlorobenzene at a concentration of 4 mg mL^−1^ and used as the active material. The solution was deposited on the PETS‐treated substrates using the solution‐shearing method. This involved placing ≈15 µL of the solution on a heated platform (80–110 °C) and controlled shearing at a rate of 40–80 µm s^−1^ using an upper shearing plate modified with octadecyltrichlorosilane (ODTS). Additionally, spin‐coated **DSpDSTQ** films were prepared as a comparison, using a concentration of 10 mg mL^−1^ and a spin rate of 2000 rpm for 60 s. Following deposition, all samples were thermally annealed at 60 °C for 1 h under vacuum. For the formation of source and drain electrodes, a 60 nm layer of Au was thermally evaporated onto the samples using a shadow mask. The channel length and width were set at 25 and 1500 µm, respectively. The electrode patterns were designed to be oriented along both the parallel and perpendicular directions with respect to the shearing direction. The electrical characteristics of the fabricated OFETs were measured by a Keithley 4200‐SCS semiconductor parameter analyzer at room temperature within an N_2_‐purged glove box.

## Conflict of Interest

The authors declare no conflict of interest.

## Supporting information

Supporting Information

## Data Availability

The data that support the findings of this study are available from the corresponding author upon reasonable request.

## References

[1] E. Lee , M. Lee , C. Park , H. Lee , J. Oh , Adv. Mater. 2017, 29, 1703638.

[2] N. Wang , A. Yang , Y. Fu , Y. Li , F. Yan , Acc. Chem. Res. 2019, 52, 277. 30620566 10.1021/acs.accounts.8b00448

[3] J. Mun , G. Wang , J. Oh , T. Katsumata , F. Lee , J. Kang , H. Wu , F. Lissel , S. Rondeau‐Gagné , J. Tok , Z. Bao , Adv. Funct. Mater. 2018, 28, 1804222.

[4] L. Janasz , M. Borkowski , P. Blom , T. Marszalek , W. Pisula , Adv. Funct. Mater. 2022, 32, 2105456.

[5] S. Fratini , M. Nikolka , A. Salleo , G. Schweicher , H. Sirringhaus , Nat. Mater. 2020, 19, 491. 32296138 10.1038/s41563-020-0647-2

[6] C. Di , H. Shen , F. Zhang , D. Zhu , Acc. Chem. Res. 2019, 52, 1113. 30908012 10.1021/acs.accounts.9b00031

[7] H. Li , W. Shi , J. Song , H. Jang , J. Dailey , J. Yu , H. Katz , Chem. Rev. 2019, 119, 3. 30403474 10.1021/acs.chemrev.8b00016

[8] J. Yang , Z. Zhao , S. Wang , Y. Guo , Y. Liu , Chem 2018, 4, 2748.

[9] S. Afraj , D. Zheng , A. Velusamy , W. Ke , S. Cuthriell , X. Zhang , Y. Chen , C. Lin , J. Ni , M. Wasielewski , W. Huang , J. Yu , C. Pan , R. Schaller , M. Chen , M. Kanatzidis , A. Facchetti , T. Marks , ACS Energy Lett. 2022, 7, 2118.

[10] C. Kuan , R. Balasaravanan , S. Hsu , J. Ni , Y. Tsai , Z. Zhang , M. Chen , E. Diau , Adv. Mater. 2023, 35, 2300681. 10.1002/adma.202300681 37029333

[11] A. Velusamy , Y. Yang , C. Lin , S. Afraj , K. Jiang , P. Chen , S. Yau , I. Osaka , S. Tung , M. Chen , C. Liu , Adv. Electron. Mater. 2022, 8, 2100648.

[12] H. Ren , J. Chen , Y. Li , J. Tang , Adv. Sci. 2021, 8, 2002418. 10.1002/advs.202002418 PMC7788634 33437578

[13] S. Kumagai , H. Ishii , G. Watanabe , C. Yu , S. Watanabe , J. Takeya , T. Okamoto , Acc. Chem. Res. 2022, 55, 660. 35157436 10.1021/acs.accounts.1c00548

[14] D. Kim , F. Kim , Chem. Mater. 2021, 33, 7572.

[15] Y. Zhang , Y. Wang , C. Gao , Z. Ni , X. Zhang , W. Hu , H. Dong , Chem. Soc. Rev. 2023, 52, 1331. 36723084 10.1039/d2cs00720g

[16] Z. Yao , J. Wang , J. Pei , Chem. Sci. 2021, 12, 1193. 10.1039/d0sc06497a PMC8179153 34163881

[17] J. Chen , W. Zhang , L. Wang , G. Yu , Adv. Mater. 2023, 35, 2210772. 10.1002/adma.202210772 36519670

[18] T. Ha , P. Sonar , B. Cobb , A. Dodabalapur , Org. Electron. 2012, 13, 136.

[19] Y. Li , B. Sun , P. Sonar , S. Singh , Org. Electron. 2012, 13, 1606.

[20] P. Sonar , T. Foong , A. Dodabalapur , Phys. Chem. Chem. Phys. 2014, 16, 4275. 24452747 10.1039/c3cp53641f

[21] T. Chen , S. Afraj , S. Hong , L. Chou , A. Velusamy , C. Chen , Y. Ezhumalai , S. Yang , I. Osaka , X. Wang , M. Chen , C. Liu , ACS Appl. Energy Mater. 2022, 5, 4149.

[22] C. Lin , A. Velusamy , S. Tung , I. Osaka , M. Chen , C. Liu , Adv. Opt. Mater. 2022, 10, 2102650.

[23] A. Velusamy , S. Afraj , S. Yau , C. Liu , Y. Ezhumalai , P. Kumaresan , M. Chen , J. Chin. Chem. Soc. 2022, 69, 1253.

[24] S. Afraj , A. Velusamy , C. Chen , J. Ni , Y. Ezhumalai , C. Pan , K. Chen , S. Yau , C. Liu , C. Chiang , C. Wu , M. Chen , J. Mater. Chem. A 2022, 10, 11254.

[25] H. Jiang , S. Zhu , Z. Cui , Z. Li , Y. Liang , J. Zhu , P. Hu , H. Zhang , W. Hu , Chem. Soc. Rev. 2022, 51, 3071. 35319036 10.1039/d1cs01136g

[26] A. Velusamy , S. Yau , C. Liu , Y. Ezhumalai , P. Kumaresan , M. Chen , J. Chin. Chem. Soc. 2023, 10.1002/jccs.202300326.

[27] S. Cheng , P. Huang , M. Ramesh , H. Chang , L. Chen , C. Yeh , C. Fung , M. Wu , C. Liu , C. Kim , H. Lin , M. Chen , C. Chu , Adv. Funct. Mater. 2014, 24, 2057.

[28] S. Vegiraju , C. Lin , P. Priyanka , D. Huang , X. Luo , H. Tsai , S. Hong , C. Yeh , W. Lien , C. Wang , S. Tung , C. Liu , M. Chen , A. Facchetti , Adv. Funct. Mater. 2018, 28, 1801025.

[29] D. Ho , S. Vegiraju , D. Choi , C. Cho , G. Kwon , P. Huang , G. Lee , T. Earmme , S. Yau , M. Chen , C. Kim , Dyes Pigm. 2019, 163, 725.

[30] W. Yi , S. Zhao , H. Sun , Y. Kan , J. Shi , S. Wan , C. Li , H. Wang , J. Mater. Chem. C 2015, 3, 10856.

[31] Z. Zeng , X. Shi , C. Chi , J. López Navarrete , J. Casado , J. Wu , Chem. Soc. Rev. 2015, 44, 6578. 25994857 10.1039/c5cs00051c

[32] P. Burrezo , J. Zafra , J. López Navarrete , J. Casado , Angew. Chem., Int. Ed. 2017, 56, 2250. 10.1002/anie.201605893 27862823

[33] J. Chen , J. Yang , Y. Guo , Y. Liu , Adv. Mater. 2022, 34, 2104325. 10.1002/adma.202104325 34605074

[34] A. Velusamy , C. Yu , S. Afraj , C. Lin , W. Lo , C. Yeh , Y. Wu , H. Hsieh , J. Chen , G. Lee , S. Tung , C. Liu , M. Chen , A. Facchetti , Adv. Sci. 2021, 8, 2002930. 10.1002/advs.202002930 PMC7788596 33437584

[35] S. Vegiraju , B. Chang , P. Priyanka , D. Huang , K. Wu , L. Li , W. Chang , Y. Lai , S. Hong , B. Yu , C. Wang , W. Chang , C. Liu , M. Chen , A. Facchetti , Adv. Mater. 2017, 29, 1702414. 10.1002/adma.201702414 28707742

[36] C. Zhang , X. Zhu , Adv. Funct. Mater. 2020, 30, 2000765.

[37] Y. Liu , J. Song , Z. Bo , Chem. Commun. 2021, 57, 302. 10.1039/d0cc07086f 33346281

[38] K. Yang , Z. Chen , Y. Wang , X. Guo , Acc. Mater. Res. 2023, 4, 237.

[39] S. N. Afraj , M. Lin , C. Wu , A. Velusamy , P. Huang , T. Peng , J. Fu , S. Tung , M. Chen , C. Liu , J. Mater. Chem. C 2022, 10, 14496.

[40] S. Vegiraju , G. He , C. Kim , P. Priyanka , Y. Chiu , C. Liu , C. Huang , J. Ni , Y. Wu , Z. Chen , G. Lee , S. Tung , C. Liu , M. Chen , A. Facchetti , Adv. Funct. Mater. 2017, 27, 1606761.

[41] C. Lin , S. N. Afraj , A. Velusamy , P. Yu , C. Cho , J. Chen , Y. Li , G. Lee , S. Tung , C. Liu , M. Chen , A. Facchetti , ACS Nano 2021, 15, 727. 33253536 10.1021/acsnano.0c07003

[42] P. Lin , Y. Shoji , S. N. Afraj , M. Ueda , C. Lin , S. Inagaki , T. Endo , S. Tung , M. Chen , C. Liu , T. Higashihara , ACS Appl. Mater. Interfaces 2021, 13, 31898. 34190528 10.1021/acsami.1c04404

[43] S. Vegiraju , X. Luo , L. Li , S. N. Afraj , C. Lee , D. Zheng , H. Hsieh , C. Lin , S. Hong , H. Tsai , G. Lee , S. Tung , C. Liu , M. Chen , A. Facchetti , Chem. Mater. 2020, 32, 1422.

[44] H. Huang , L. Yang , A. Facchetti , T. Marks , Chem. Rev. 2017, 117, 10291. 28671815 10.1021/acs.chemrev.7b00084

[45] P. Lin , S. Inagaki , J. Liu , M. Chen , T. Higashihara , C. Liu , Chem. Eng. J. 2023, 458, 141366.

[46] S. Vegiraju , A. Amelenan Torimtubun , P. Lin , H. Tsai , W. Lien , C. Chen , G. He , C. Lin , D. Zheng , Y. Huang , Y. Wu , S. Yau , G. Lee , S. Tung , C. Wang , C. Liu , M. Chen , A. Facchetti , ACS Appl. Mater. Interfaces 2020, 12, 25081. 32340439 10.1021/acsami.0c03477

[47] Z. Shan , J. Shi , W. Xu , C. Li , H. Wang , Dyes Pigm. 2019, 171, 107675.

[48] Q. Liu , S. Kumagai , S. Manzhos , Y. Chen , I. Angunawela , M. Nahid , K. Feron , S. Bottle , J. Bell , H. Ade , J. Takeya , P. Sonar , Adv. Funct. Mater. 2020, 30, 2000489.

[49] J. Hollinger , D. Gao , D. Seferos , Isr. J. Chem. 2014, 54, 440.

[50] Z. Fei , Y. Han , E. Gann , T. Hodsden , A. Chesman , C. Mcneill , T. Anthopoulos , M. Heeney , J. Am. Chem. Soc. 2017, 139, 8552. 28548496 10.1021/jacs.7b03099

[51] S. Jang , I. Kim , M. Kang , Z. Fei , E. Jung , T. Mccarthy‐Ward , J. Shaw , D. Lim , Y. Kim , S. Mathur , M. Heeney , D. Kim , Adv. Sci. 2019, 6, 1900245. 10.1002/advs.201900245 PMC6662335 31380184

[52] M. Sung , A. Luzio , W. Park , R. Kim , E. Gann , F. Maddalena , G. Pace , Y. Xu , D. Natali , C. De Falco , L. Dang , C. Mcneill , M. Caironi , Y. Noh , Y. Kim , Adv. Funct. Mater. 2016, 26, 4984.

[53] Z. Ma , C. Udamulle Gedara , H. Wang , M. Biewer , M. Stefan , ACS Appl. Mater. Interfaces 2023, 15, 46119. 37738113 10.1021/acsami.3c09130

[54] Q. Liu , Y. Wang , A. Kohara , H. Matsumoto , S. Manzhos , K. Feron , S. E. Bottle , J. Bell , T. Michinobu , P. Sonar , Adv. Funct. Mater. 2020, 30, 1907452.

[55] S. Afraj , C. Lin , A. Velusamy , C. Cho , H. Liu , J. Chen , G. Lee , J. Fu , J. Ni , S. Tung , S. Yau , C. Liu , M. Chen , A. Facchetti , Adv. Funct. Mater. 2022, 32, 2200880.

[56] S. Handa , E. Miyazaki , K. Takimiya , Chem. Commun. 2009, 3919. 10.1039/b905472c 19662252

[57] Y. Getmanenko , T. Purcell , D. Hwang , B. Kippelen , S. Marder , J. Org. Chem 2012, 77, 10931. 23167502 10.1021/jo3020006

[58] C. Wang , M. Abbas , G. Wantz , K. Kawabata , K. Takimiya , J. Mater. Chem. C 2020, 8, 15119.

[59] H. Takenaka , T. Ogaki , C. Wang , K. Kawabata , K. Takimiya , Chem. Mater. 2019, 31, 6696.

[60] T. Okamoto , M. Mitani , C. Yu , C. Mitsui , M. Yamagishi , H. Ishii , G. Watanabe , S. Kumagai , D. Hashizume , S. Tanaka , M. Yano , T. Kushida , H. Sato , K. Sugimoto , T. Kato , J. Takeya , J. Am. Chem. Soc. 2020, 142, 14974. 32812421 10.1021/jacs.0c05522

[61] P. Gamage , C. Udamulle Gedara , Z. Ma , A. Bhadran , R. Gunawardhana , C. Bulumulla , M. Biewer , M. Stefan , ACS Appl. Electron. Mater. 2021, 3, 5335.

[62] G. Barbarella , L. Favaretto , G. Sotgiu , M. Zambianchi , C. Arbizzani , A. Bongini , M. Mastragostino , Chem. Mater. 1999, 11, 2533.

[63] Q. Wu , R. Li , W. Hong , H. Li , X. Gao , D. Zhu , Chem. Mater. 2011, 23, 3138.

[64] Q. Wu , X. Qiao , Q. Huang , J. Li , Y. Xiong , X. Gao , H. Li , RSC Adv. 2014, 4, 16939.

[65] G. Chen , H. Sasabe , W. Lu , X. Wang , J. Kido , Z. Hong , Y. Yang , J. Mater. Chem. C 2013, 1, 6547.

[66] J. H. Kim , T. Schembri , D. Bialas , M. Stolte , F. Würthner , Adv. Mater. 2022, 34, 2104678. 10.1002/adma.202104678 34668248

[67] M. Más‐Montoya , R. Janssen , Adv. Funct. Mater. 2017, 27, 1605779.

[68] W. Chen , W. Shen , H. Wang , F. Liu , L. Duan , X. Xu , D. Zhu , M. Qiu , E. Wang , R. Yang , Dyes Pigm. 2019, 166, 42.

[69] E. D. Glowacki , L. Leonat , G. Voss , M. Bodea , Z. Bozkurt , A. Ramil , M. Irimia‐Vladu , S. Bauer , N. Sariciftci , AIP Adv. 2011, 1, 042132.

[70] Y. Zhang , L. Kong , Y. Zhang , H. Du , J. Zhao , S. Chen , Y. Xie , Y. Wang , Org. Electron. 2020, 81, 105685.

[71] S. Martens , U. Zschieschang , H. Wadepohl , H. Klauk , L. Gade , Chem. Eur. J 2012, 18, 3498. 22354835 10.1002/chem.201103158

[72] B. Jones , M. Ahrens , M. Yoon , A. Facchetti , T. Marks , M. Wasielewski , Angew. Chem., Int. Ed. 2004, 43, 6363. 10.1002/anie.200461324 15558692

[73] J. H. Oh , S. Liu , Z. Bao , R. Schmidt , F. Würthner , Appl. Phys. Lett. 2007, 91, 212107.

[74] S. MM , Adv. Mater 2007, 19, 3692.

[75] S. Debnath , S. Chithiravel , S. Sharma , A. Bedi , K. Krishnamoorthy , S. Zade , ACS Appl. Mater. Interfaces 2016, 8, 18222. 27353123 10.1021/acsami.6b02154

[76] M. Jeffries‐El , B. Kobilka , B. Hale , Macromolecules 2014, 47, 7253.

[77] S. Zade , N. Zamoshchik , M. Bendikov , Chem. ‐ Eur. J. 2009, 15, 8613. 19658134 10.1002/chem.200900971

[78] P. Ou , W. Shen , R. He , X. Xie , C. Zeng , M. Li , Polym. Int. 2011, 60, 1408.

[79] H. Becerril , M. Roberts , Z. Liu , J. Locklin , Z. Bao , Adv. Mater. 2008, 20, 2588.

[80] Y. Yin , S. Zhu , S. Chen , Z. Lin , J. Peng , Adv. Funct. Mater. 2022, 32, 2110824.

[81] X. Gu , L. Shaw , K. Gu , M. Toney , Z. Bao , Nat. Commun. 2018, 9, 534. 29416035 10.1038/s41467-018-02833-9 PMC5803241

[82] G. Giri , E. Verploegen , S. Mannsfeld , S. Atahan‐Evrenk , D. Kim , S. Lee , H. Becerril , A. Aspuru‐Guzik , M. Toney , Z. Bao , Nature 2011, 480, 504. 22193105 10.1038/nature10683

[83] M. Shahid , T. Mccarthy‐Ward , J. Labram , S. Rossbauer , E. Domingo , S. Watkins , N. Stingelin , T. Anthopoulos , M. Heeney , Chem. Sci. 2012, 3, 181.

[84] S. Ray , J. Panidi , T. Mukhopadhyay , U. Salzner , T. Anthopoulos , S. Patil , ACS Appl. Electron. Mater. 2019, 1, 2037.

[85] E. Smits , T. Anthopoulos , S. Setayesh , E. Van Veenendaal , R. Coehoorn , P. Blom , B. De Boer , D. De Leeuw , Phys. Rev. B 2006, 73, 205316.

[86] E. Meijer , D. De Leeuw , S. Setayesh , E. Van Veenendaal , B. Huisman , P. Blom , J. Hummelen , U. Scherf , T. Klapwijk , Nat. Mater. 2003, 2, 678. 14502272 10.1038/nmat978

[87] S. Kim , H. Yoo , J. Choi , Sensors 2023, 23, 2265. 36850862 10.3390/s23042265 PMC9959125

[88] T. Yu , Z. Liu , Y. Wang , L. Zhang , S. Hou , Z. Wan , J. Yin , X. Gao , L. Wu , Y. Xia , Z. Liu , Sci. Rep. 2023, 13, 5865. 37041232 10.1038/s41598-023-32959-w PMC10090149

[89] R. Schroeder , L. Majewski , M. Grell , Appl. Phys. Lett. 2003, 83, 3201.

[90] Z. Chen , M. Bird , V. Lemaur , G. Radtke , J. Cornil , M. Heeney , I. Mcculloch , H. Sirringhaus , Phys. Rev. B 2011, 84, 115211.

[91] M. Gwinner , Y. Vaynzof , K. Banger , P. Ho , R. Friend , H. Sirringhaus , Adv. Funct. Mater. 2010, 20, 3457.

[92] J. Deng , Y. Xu , L. Liu , C. Feng , J. Tang , Y. Gao , Y. Wang , B. Yang , P. Lu , W. Yang , Y. Ma , Chem. Commun. 2016, 52, 2370. 10.1039/c5cc09702a 26730680

[93] M. Nakano , I. Osaka , K. Takimiya , Adv. Mater. 2017, 29, 1602893. 10.1002/adma.201602893 28042890

[94] R. Rogowski , A. Darhuber , Langmuir 2010, 26, 11485. 20486716 10.1021/la101002x

[95] J. Rivnay , R. Noriega , R. Kline , A. Salleo , M. Toney , Phys. Rev. B 2011, 84, 045203.

