# Supplementary material for: Diselenophene‐Dithioalkylthiophene Based Quinoidal Small Molecules for Ambipolar Organic Field Effect Transistors
Source: Adv Sci (Weinh). 2023 Dec 14;11(9):2305361. doi: 10.1002/advs.202305361 (PMC10916611; doi:10.1002/advs.202305361)
Supplement: Supplementary file 1 — Supporting Information [file ADVS-11-2305361-s001.pdf]

ADVANCED SCIENCE
Open Access

Supporting Information

for *Adv. Sci.*, DOI 10.1002/advs.202305361

Diselenophene-Dithioalkylthiophene Based Quinoidal Small Molecules for Ambipolar Organic Field Effect Transistors

Arulmozhi Velusamy, Yen-Yu Chen, Meng-Hao Lin, Shakil N. Afraj, Jia-Hao Liu, Ming-Chou Chen and Cheng-Liang Liu**

Supporting Information

Diselenophene-dithioalkylthiophene Based Quinoidal Small Molecules for Ambipolar Organic Field Effect Transistors

Arulmozhi Velusamy, Yen-Yu Chen, Meng-Hao Lin, Shakil N. Afraj, Jia-Hao Liu, Ming-Chou Chen, and Cheng-Liang Liu**

Dr. A. Velusamy, Dr. S. N. Afraj, J.-H. Liu, Prof. M.-C. Chen
Department of Chemistry and Research Center of New Generation Light Driven Photovoltaic Modules
National Central University
Taoyuan, 32001 Taiwan
E-mail: mcchen@ncu.edu.tw

Y.-Y. Chen, M.-H. Lin, Prof. C.-L. Liu
Department of Materials Science and Engineering
National Taiwan University
Taipei 10617, Taiwan
E-mail: liucl@ntu.edu.tw

Experimental Section

Characterization

^1H and ^{13}C NMR spectra were recorded using a Bruker 500 or a 300 instrument, with reference to solvent signals. Differential scanning calorimetry (DSC) was carried out on a Mettler DSC 822 instrument at a scan rate of 10 K/min. Thermo gravimetric analysis (TGA) was performed on a Perkin Elmer TGA-7 thermal analysis system using dry nitrogen as a carrier gas at a flow rate of 40 mL/min. The UV–vis spectrum was characterized with a JASCO V-670 UV–vis spectrophotometer. Differential pulse voltammetry experiments were performed with a conventional three-electrode configuration (a platinum disk working electrode, an auxiliary platinum wire electrode, and a non-aqueous Ag reference electrode, with a supporting electrolyte of 0.1 M tetrabutylammonium hexafluorophosphate (dry $TBAPF_6$) in the specified dry solvent, using a CHI621C Electrochemical Analyzer (CH Instruments). Under N_2, the anhydrous OSC material was dissolved in above 0.1 M *o*-$C_6H_4Cl_2$ solution to prepare a 10^{-3} M test solution. In each DPV experiment, 5 mL of the test solution is scanned together with Fc/Fc^+ (also 10^{-3} M; as internal standard) under N_2. electrochemical potentials were referenced to an Fc/Fc^+ internal standard (at +0.6 V). Mass spectrometric data were obtained with an ATS-00670 HRMS instrument.

Materials

Starting materials (from Sigma Aldrich, Alfa, or TCI Chemical Co.) were reagent grade and were used without further purification unless otherwise indicated. Reaction solvents (toluene, and tetrahydrofuran) were distilled under nitrogen from sodium/benzophenone ketyl, and halogenated solvents were distilled from CaH_2. Compounds 3,4-dibromothiophene (**4**) to 2,5-dibromo-3,4-bis(alkylthio)thiophene (**7a-c**) and tributyl(selenophen-2-yl)stannane were synthesized as reported previously.[1-2]

General procedure for the synthesis of 8a-c

Under the anhydrous condition, $Pd(PPh_3)_4$ (0.05 equiv) was added to a solution of 2,5-dibromo-3,4- bis(alkylthio)thiophene (7a-c; 1 equiv) and mono-stannylated selenophene (2.5 equiv) in dry toluene. The resulting mixture was refluxed for 24 hours under nitrogen. After cooling to room temperature, the solvent was evaporated and the obtained residue was purified by column chromatography with hexanes as the eluent.

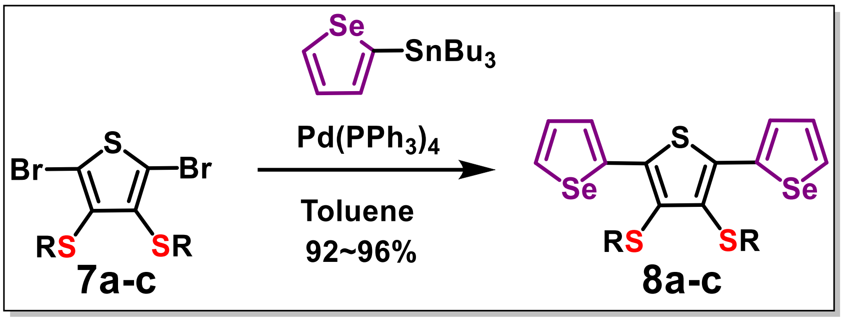

Scheme S1. Synthesis of central core **DSpDST** (**8a-c**).

Synthesis of 8a: The title compound was obtained as a pale yellow liquid, (yield = 93%). ^{1}H NMR (500 MHz, $CDCl_3$): δ 8.05 (d, J = 6 Hz, 2H), 7.63 (d, J = 4 Hz, 2H), 7.29 (dd, J = 5.5 Hz, 2H), 2.87 (t, J = 7.2 Hz, 4H), 1.63-1.57 (m, 4H), 1.36-1.33 (m, 4H), 1.29-1.22 (m, 8H), 0.85 (t, J = 6.9 Hz, 6H).

Synthesis of 8b: The title compound was obtained as a pale yellow liquid, (yield = 92%). ^{1}H NMR (500 MHz, $CDCl_3$): δ 8.05 (d, J = 6 Hz, 2H), 7.63 (d, J = 3.9 Hz, 2H), 7.29 (dd, J = 4 Hz, 2H), 2.87 (t, J = 7.4 Hz, 4H), 1.63-1.57 (m, 4H), 1.38-1.33 (m, 4H), 1.29-1.22 (m, 24H), 0.87 (t, J = 6.9 Hz, 6H).

Synthesis of 8c: The title compound was obtained as a pale yellow liquid, (yield = 96%). ^{1}H NMR (500 MHz, $CDCl_3$): δ 8.05 (d, J = 5.8 Hz, 2H), 7.62 (d, J = 3.9 Hz, 2H), 7.29 (dd, J = 5.5 Hz, 2H), 2.87 (t, J = 7.4 Hz, 4H), 1.64-1.58 (m, 4H), 1.38-1.33 (m, 4H), 1.25-1.22 (m, 40H), 0.87 (t, J = 6.8 Hz, 6H).

General procedure for the synthesis of 9a-c

At 0 °C, NBS (2.2 equiv) was added to a 30 mL DMF solution of 3',4'-bis(alkylthio)-2,2':5',2"-S7 terthiophene (8a-c) (1 equiv), and the mixture was stirred for 30 min, warmed to room temperature, and stirred for 12 h. Next, water was added to this solution, and the desired product was extracted with ether and washed with brine, dried over Na_2SO_4. The solvent was evaporated and the obtained residue was purified by column chromatography with hexanes as the eluent.

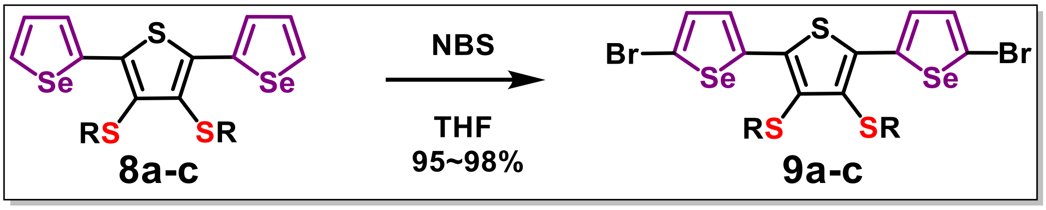

Scheme S2. Synthesis of intermediate compound **9a-c**.

Synthesis of 9a: The title compound was obtained as a yellow solid, (yield = 98%). (300 MHz, $CDCl_3$): δ 7.29 (d, J = 4.3 Hz, 2 H), 7.21 (d, J = 4.3 Hz, 2 H), 2.87 (t, J = 7.5 Hz, 4 H), 1.66-1.56 (m, 4 H), 1.42-1.36 (m, 4 H), 1.27-1.25 (m, 8 H), 0.86 (t, J = 6.7 Hz, 6H).

Synthesis of 9b: The title compound was obtained as a yellow solid, (yield = 95%). (500 MHz, $CDCl_3$): δ 7.29 (d, J = 4.4 Hz, 2 H), 7.21 (d, J = 4.4 Hz, 2 H), 2.87 (t, J = 7.4 Hz, 4 H), 1.64-1.58 (m, 4 H), 1.39-1.34 (m, 4 H), 1.30-1.24 (m, 24 H), 0.87 (t, J = 6.9 Hz, 6H).

Synthesis of 9c: The title compound was obtained as a yellow solid, (yield = 97%). (500 MHz, $CDCl_3$): δ 7.29 (d, J = 4.3 Hz, 2 H), 7.21 (d, J = 4.3 Hz, 2 H), 2.87 (t, J = 7.5 Hz, 4 H), 1.66-1.56 (m, 4 H), 1.38-1.34 (m, 4 H), 1.31-1.23 (m, 40 H), 0.87 (t, J = 6.9 Hz, 6H).

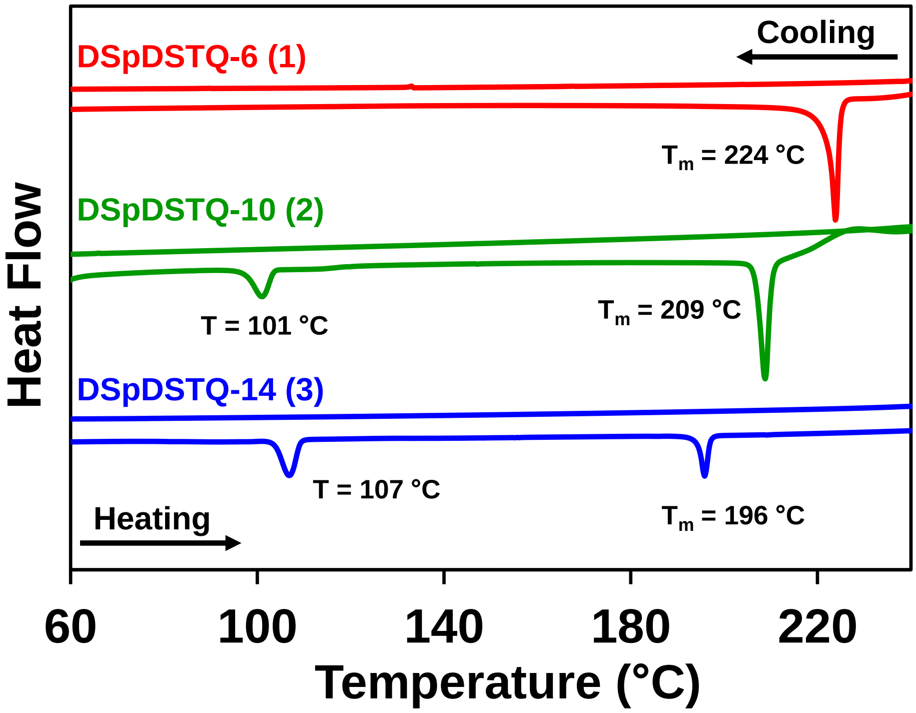

Figure S1. DSC curves of **DSpDSTQ** (**1**–**3**).

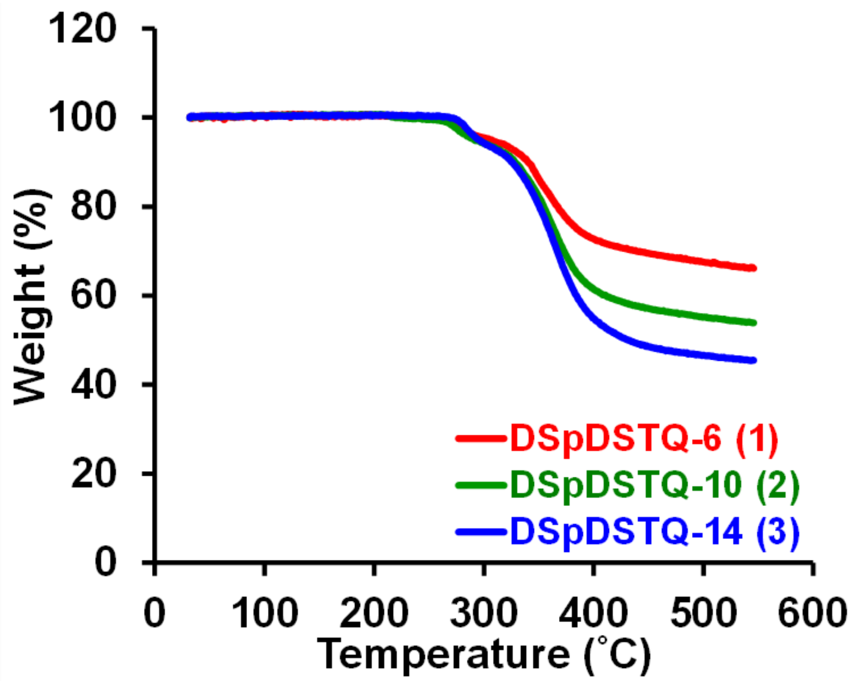

Figure S2. TGA curves of **DSpDSTQ** (**1**–**3**).

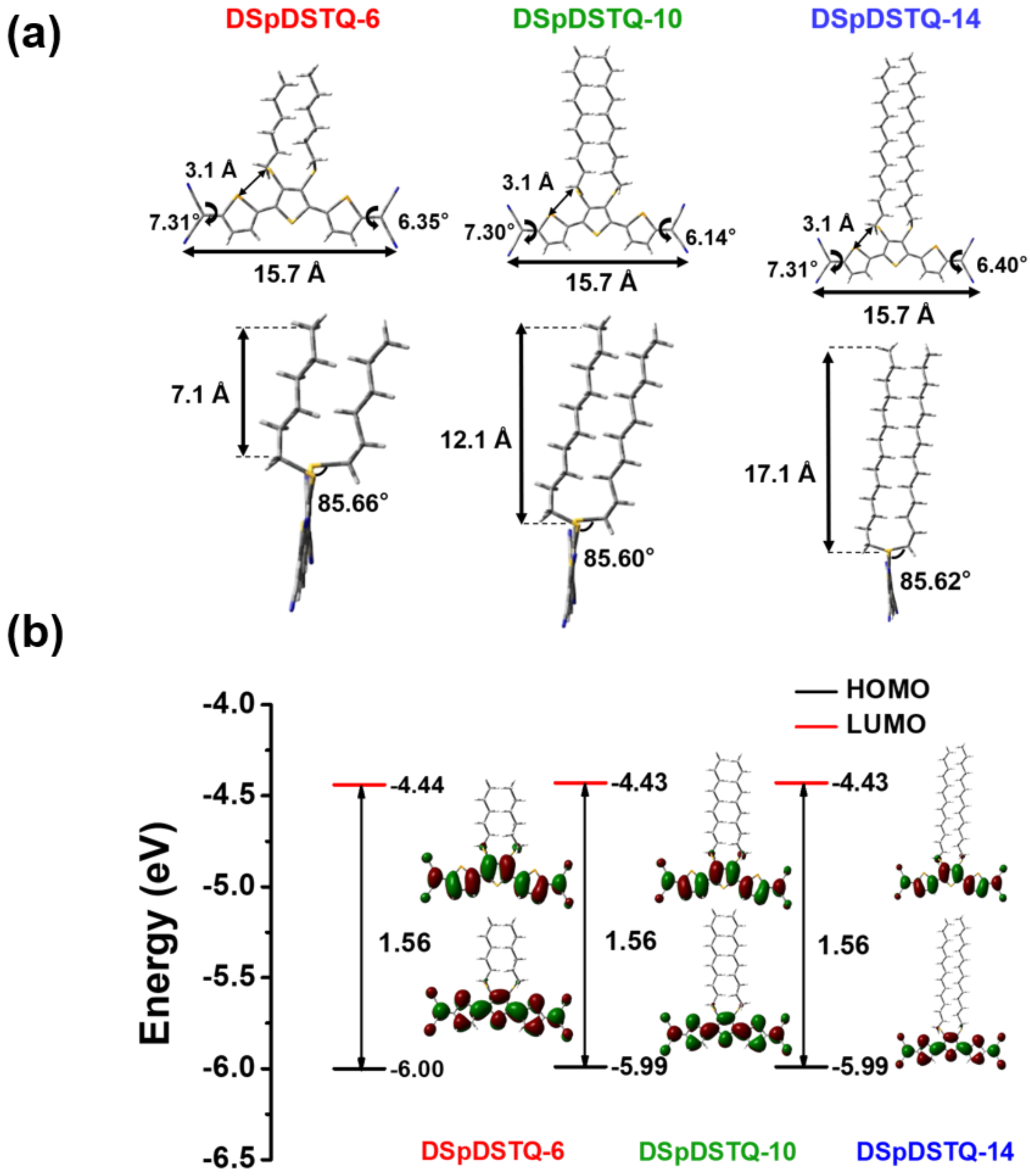

Figure S3. DFT calculation of **DSpDSTQ** derivatives: (a) optimized geometries and (b) energy levels.

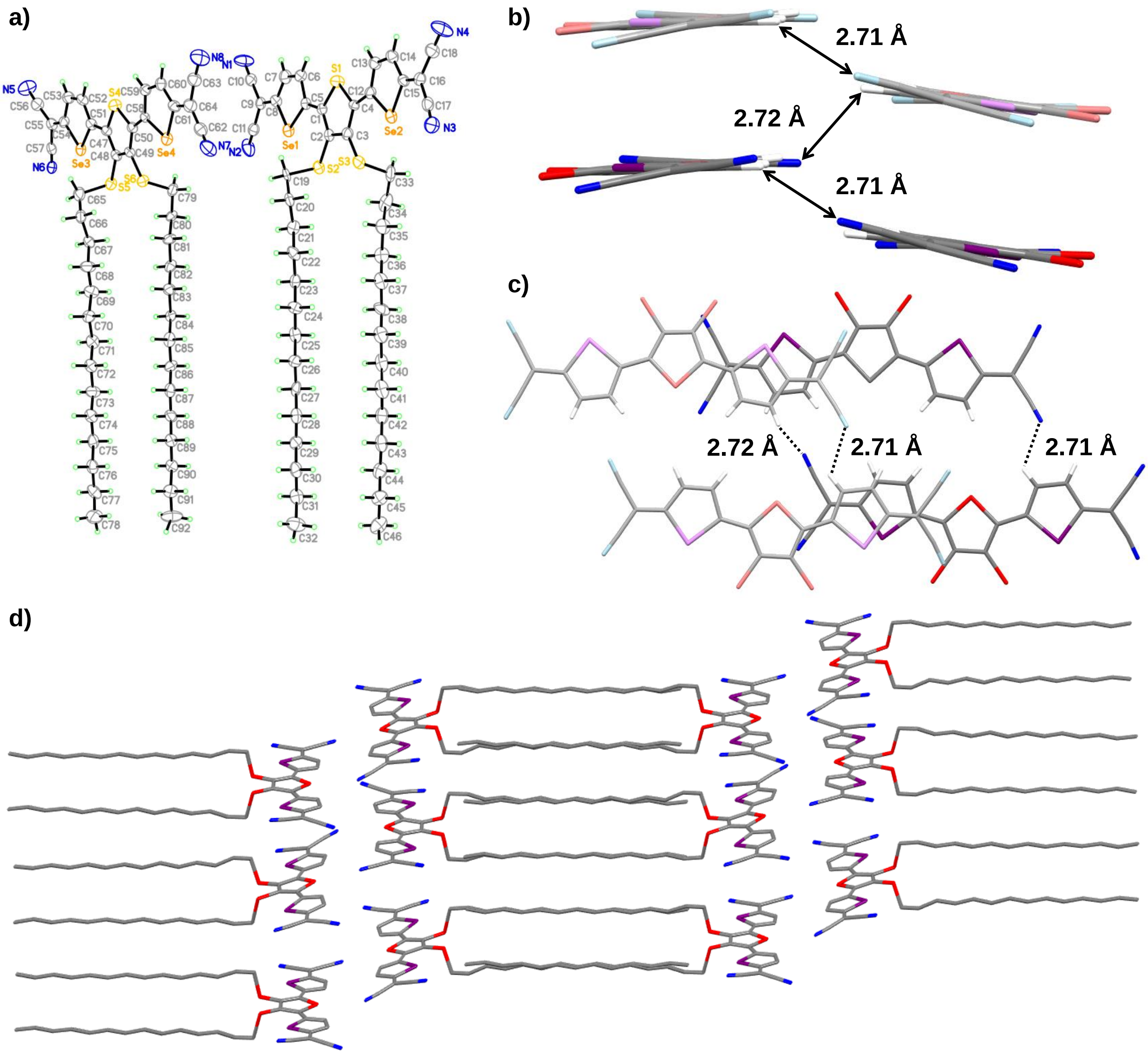

Figure S4. a) Molecular structure of **3** by single crystal X-ray analysis. (b-c) Side and top views of the molecular packing of **DSpDSTQ-14** (**3**) in stick models with a short intermolecular N−H distance of ~2.72 Å. d) Face-to-face slipped π−π stacking arrangement of **DSpDSTQ-14** (**3**). The selenium, sulfur, and nitrogen atoms are specified in purple, red, and blue color, respectively. The alkyl chains are omitted for clarity.

Table S1. Summary of crystal structure data for **DSpDSTQ-14** (**3**).

Identification code	ic21624	
Empirical formula	C93 H126 Cl2 N8 S6 Se4	
Formula weight	1935.11	
Temperature	200(2) K	
Wavelength	0.71073 Å	
Crystal system	Triclinic	
Space group	P-1	
Unit cell dimensions	a = 9.6879(13) Å	□= 96.281(5)°.
	b = 15.4153(19) Å	□= 91.681(5)°.
	c = 31.785(4) Å	□ = 92.273(5)°.
Volume	4712.0(11) Å^3	
Z	2	
Density (calculated)	1.364 Mg/m^3	
Absorption coefficient	1.795 mm^{-1}	
F(000)	2012	
Crystal size	0.504 x 0.216 x 0.055 mm^3	
Theta range for data collection	1.935 to 25.393°.	
Index ranges	-11<=h<=8, -18<=k<=18, -37<=l<=38	
Reflections collected	45075	
Independent reflections	17209 [R(int) = 0.0886]	
Completeness to theta = 25.242°	99.6 %	
Absorption correction	Semi-empirical from equivalents	
Max. and min. transmission	0.8620 and 0.6873	
Refinement method	Full-matrix least-squares on F^2	
Data / restraints / parameters	17209 / 233 / 1023	
Goodness-of-fit on F^2	1.042	
Final R indices [I>2sigma(I)]	R1 = 0.1174, wR2 = 0.3086	
R indices (all data)	R1 = 0.1837, wR2 = 0.3636	
Extinction coefficient	n/a	
Largest diff. peak and hole	1.914 and -1.199 e.Å^{-3}	

Crystallographic data (excluding structure factors) for the structure(s) reported in this paper have been deposited with the Cambridge Crystallographic Data Centre as supplementary publication no. **CCDC 2257869**

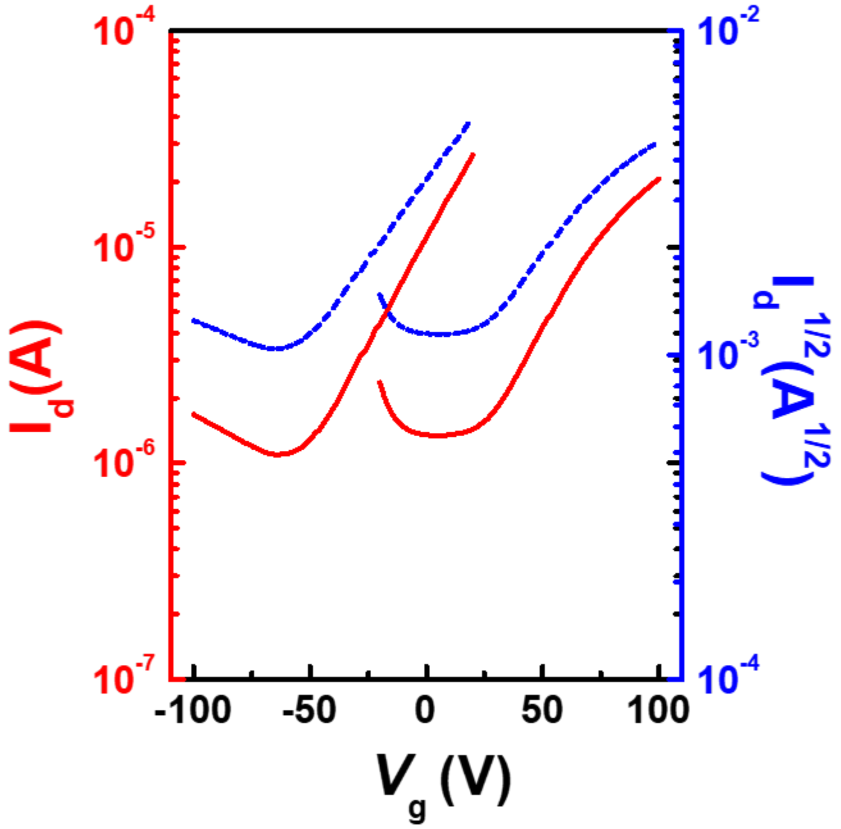

Figure S5. Representative transfer characteristics of spin-coated OFETs based on **DSpDSTQ-6** thin films with a constant V_d of 100 V for n-type and –100 V for p-type measurements.

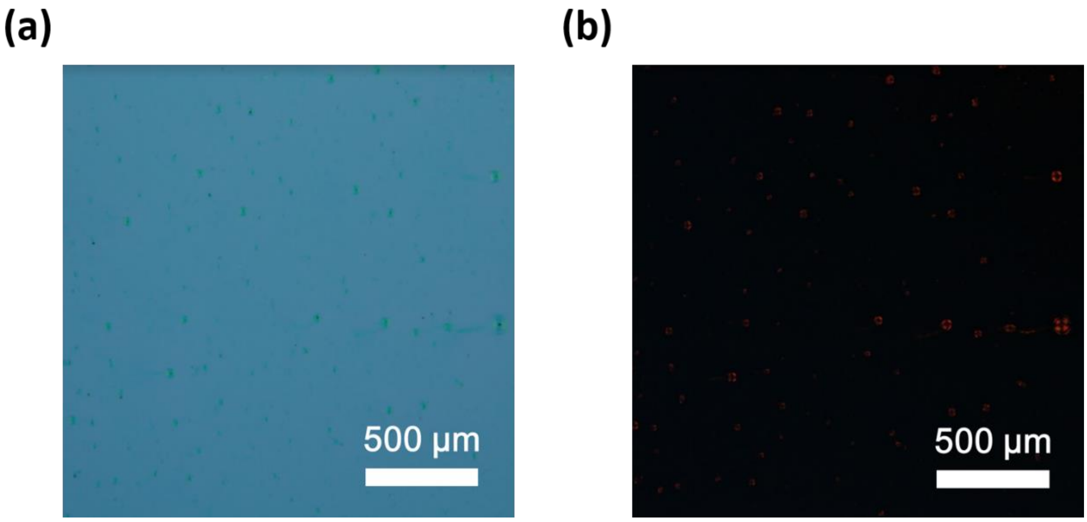

Figure S6. DSpDSTQ-6 spin-coated (2000 rpm, 60s) thin film (a) OM (b) POM images.

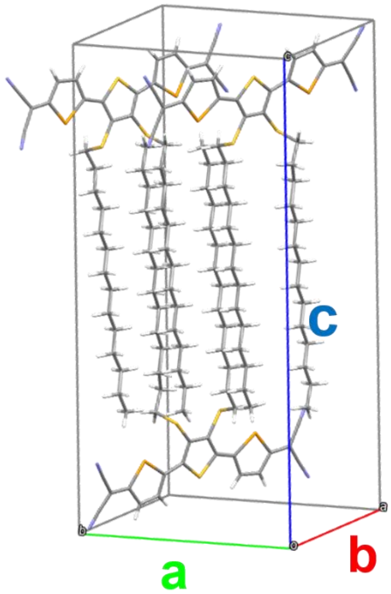

Figure S7. Single crystal derived unit cell of **DSpDSTQ-14**.

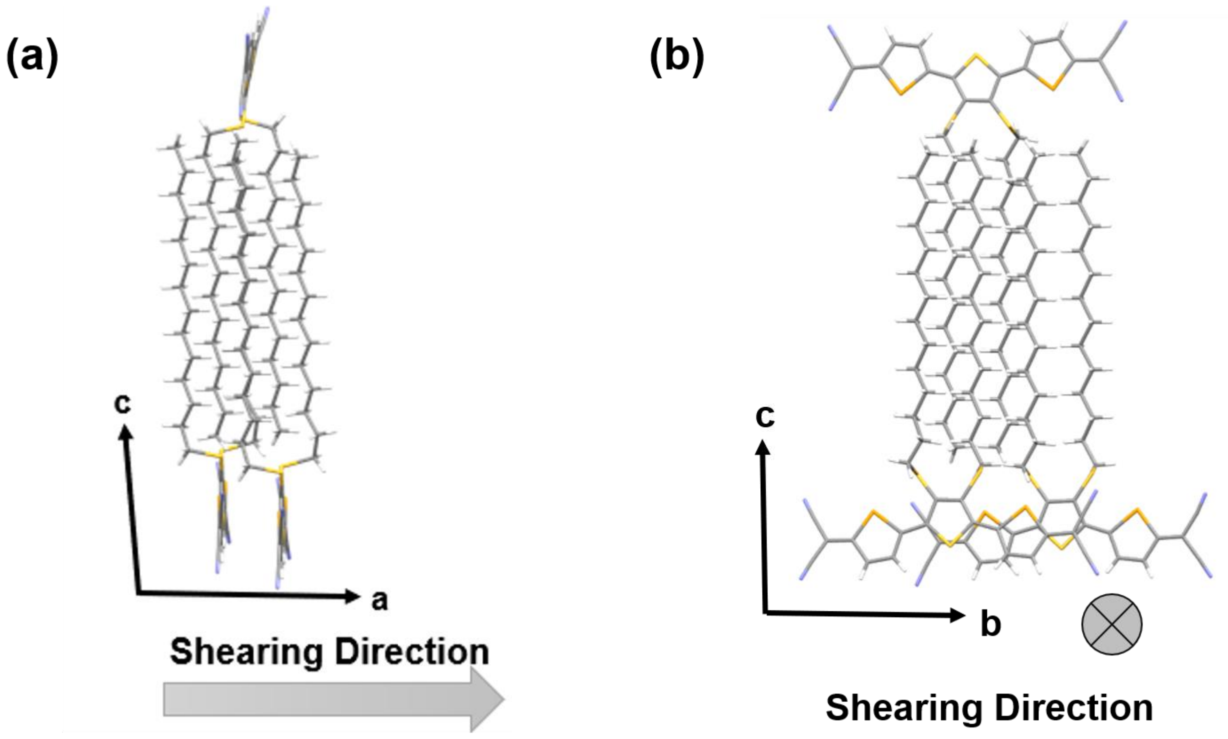

Figure S8. The schematic diagram of molecular packing of **DSpDSTQ-14** from (a) parallel and (b) perpendicular incident beam direction compared with shearing direction.

Table S2. Lattice parameters of **DSpDSTQ** solution-sheared films derived from GIXRD measurements.

**Compounds**	c [Å]		b [Å]		a [Å]	
Shearing Direction	//	⊥	//	⊥	//	⊥
**DSpDSTQ-6 (1)**	23.8	23.4	10.6	-	-	11.9
**DSpDSTQ-10 (2)**	26.8	26.4	-	-	-	12.9
**DSpDSTQ-14 (3)**	29.4	30.1	-	-	-	13.0

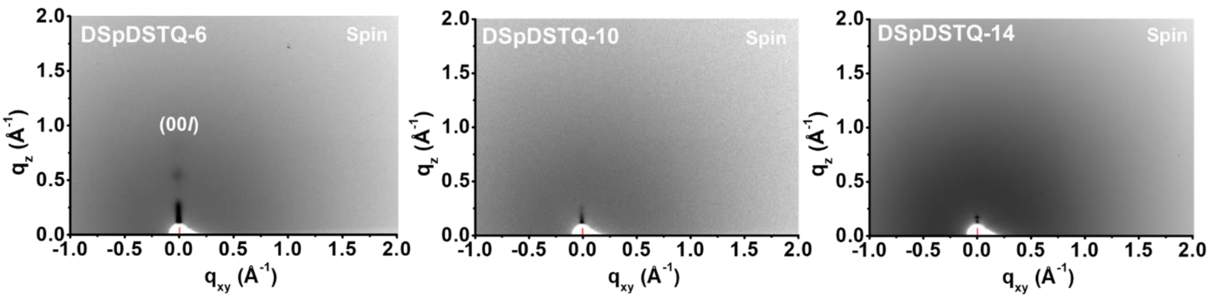

Figure S9. 2D GIXRD patterns of spin-coated **DSpDSTQ-6**, **DSpDSTQ-10**, and **DSpDSTQ-14** films.

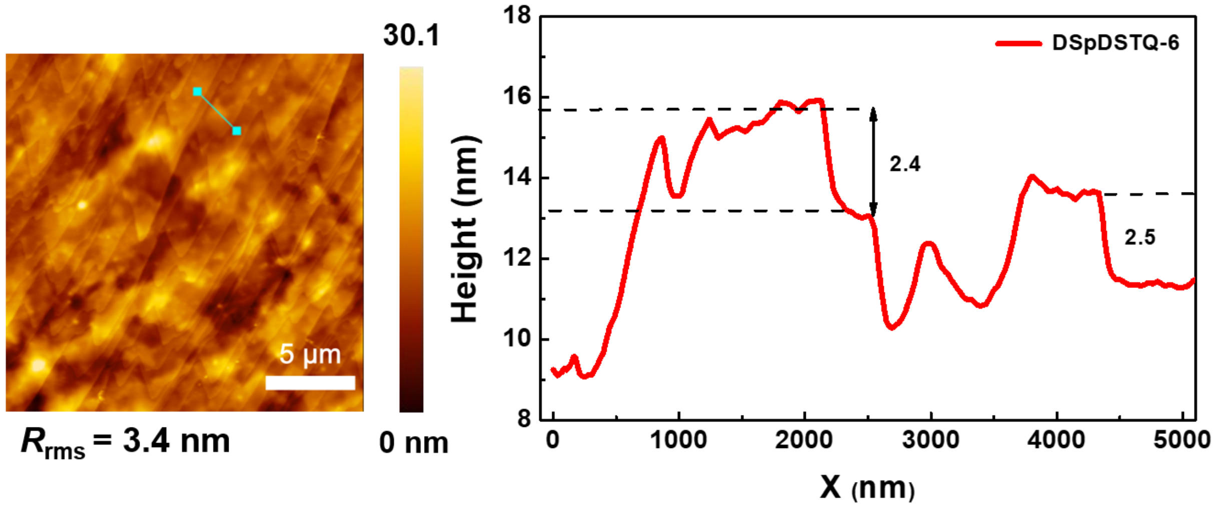

Figure S10. AFM images and height profiles (along the line) of solution-sheared **DSpDSTQ-6**.

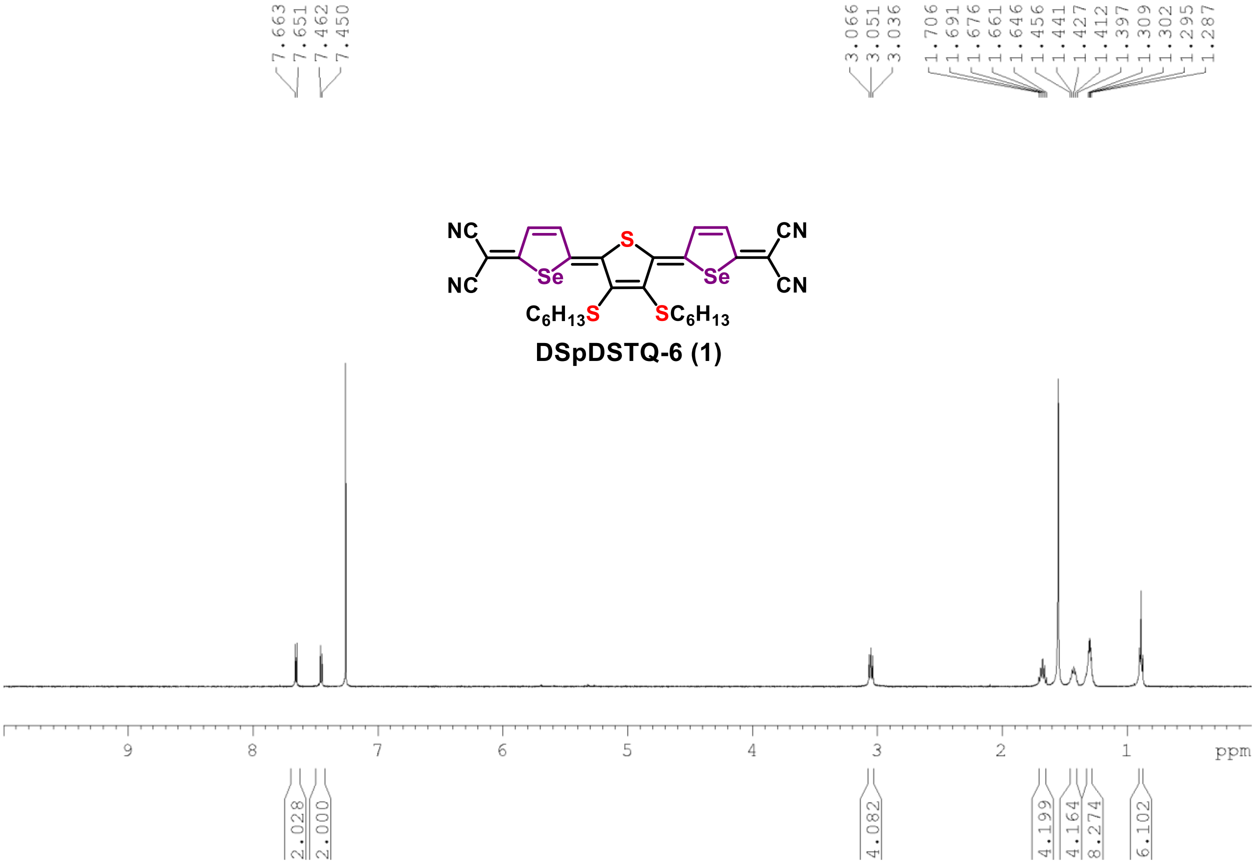
7.663
7.651
7.462
7.450
3.066
3.051
3.036
1.706
1.691
1.676
1.661
1.646
1.456
1.441
1.427
1.412
1.397
1.309
1.302
1.295
1.287
DSpDSTQ-6 (1)
ppm
2.028
2.000
4.082
4.199
4.164
8.274
6.102

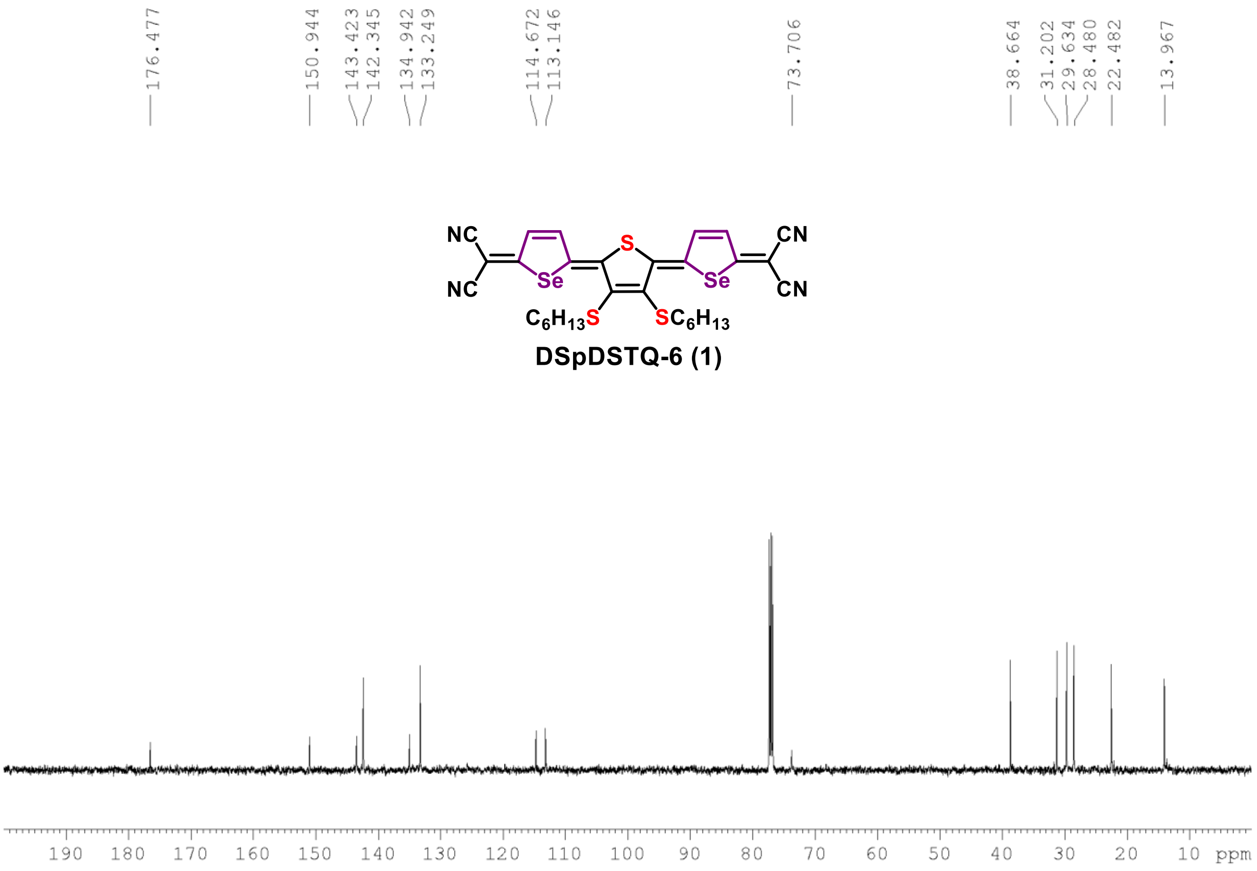
176.477
150.944
143.423
142.345
134.942
133.249
114.672
113.146
73.706
38.664
31.202
29.634
28.480
22.482
13.967
DSpDSTQ-6 (1)
ppm

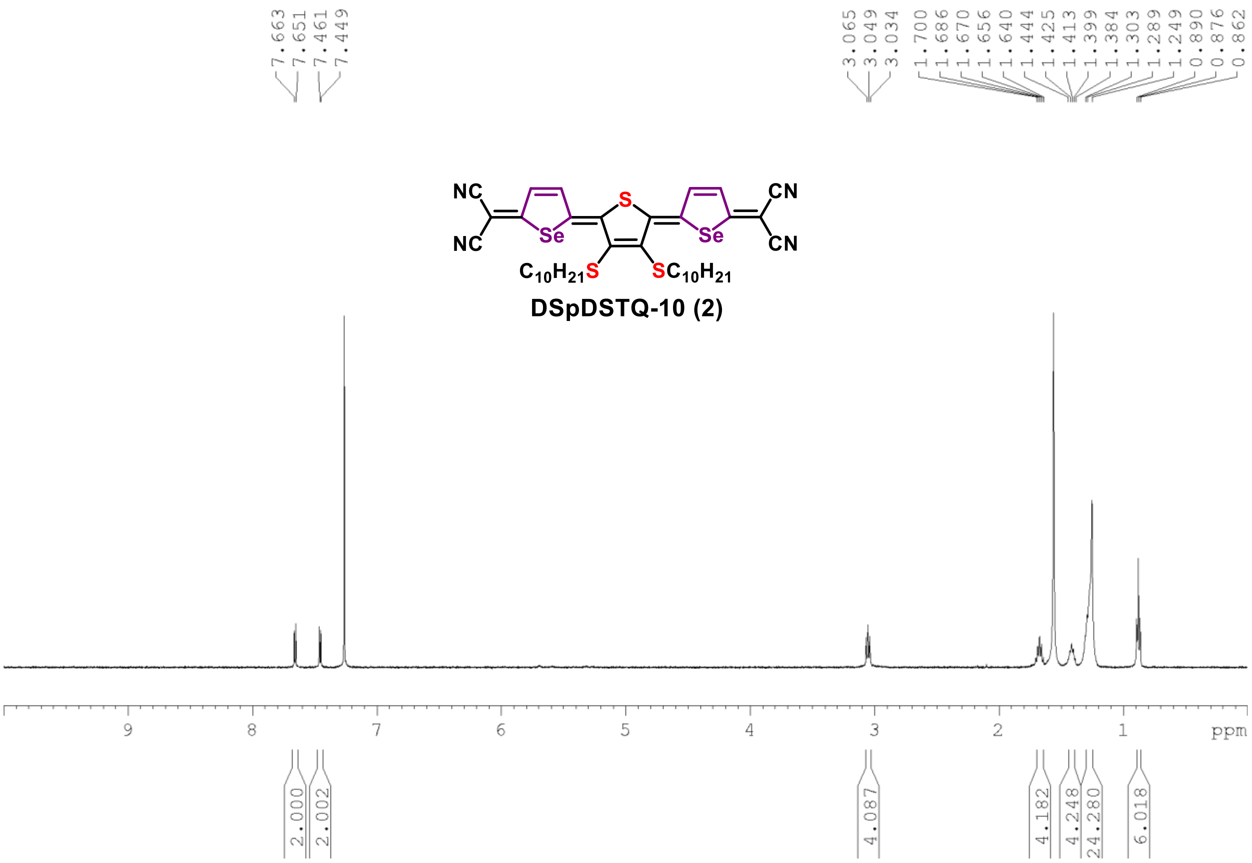
7.663
7.651
7.461
7.449
3.065
3.049
3.034
1.700
1.686
1.670
1.656
1.640
1.444
1.425
1.413
1.399
1.384
1.303
1.289
1.249
0.890
0.876
0.862
NC
NC
Se
S
Se
CN
CN
C10H21S
SC10H21
DSpDSTQ-10 (2)
9
8
7
6
5
4
3
2
1
ppm
2.000
2.002
4.087
4.182
4.248
24.280
6.018

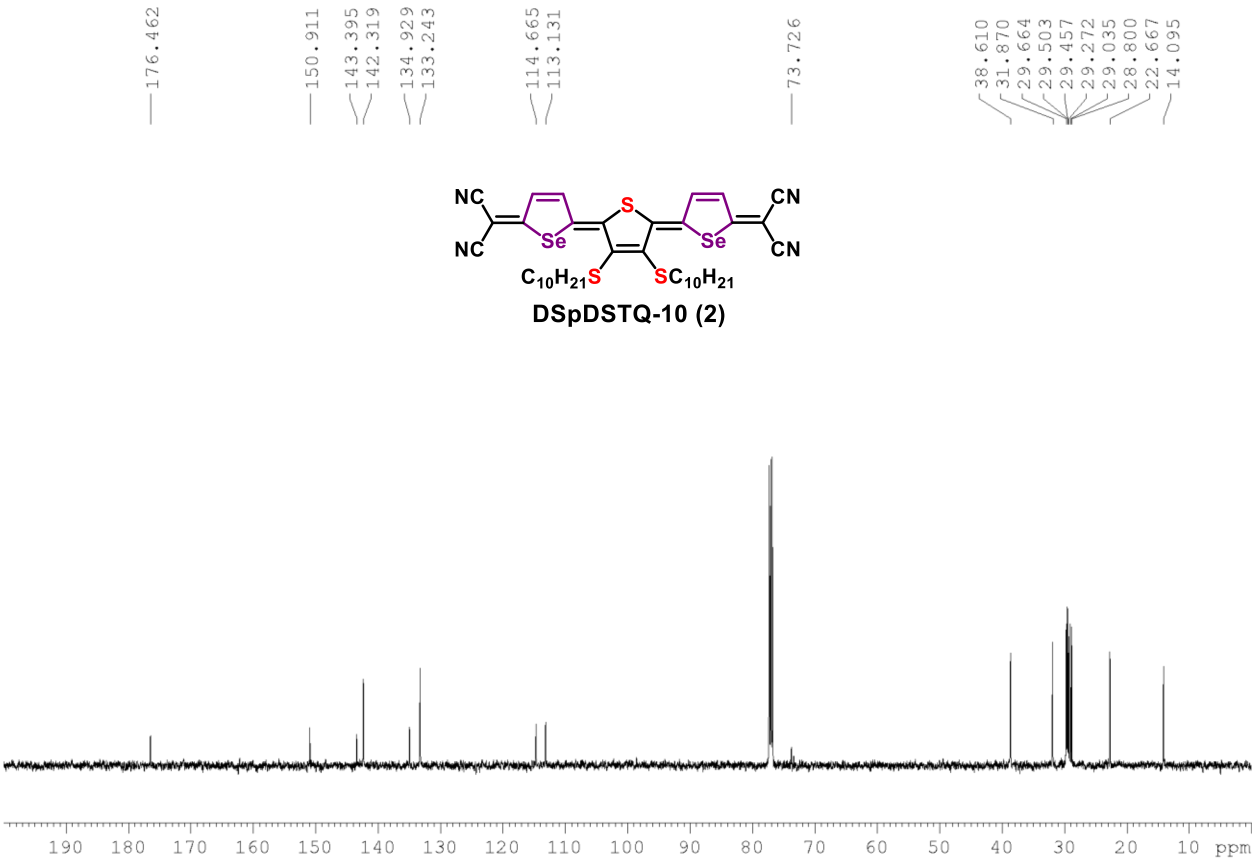
176.462
150.911
143.395
142.319
134.929
133.243
114.665
113.131
73.726
38.610
31.870
29.664
29.503
29.457
29.272
29.035
28.800
22.667
14.095
NC
NC
Se
S
Se
CN
CN
C10H21S
SC10H21
DSpDSTQ-10 (2)
190
180
170
160
150
140
130
120
110
100
90
80
70
60
50
40
30
20
10
ppm

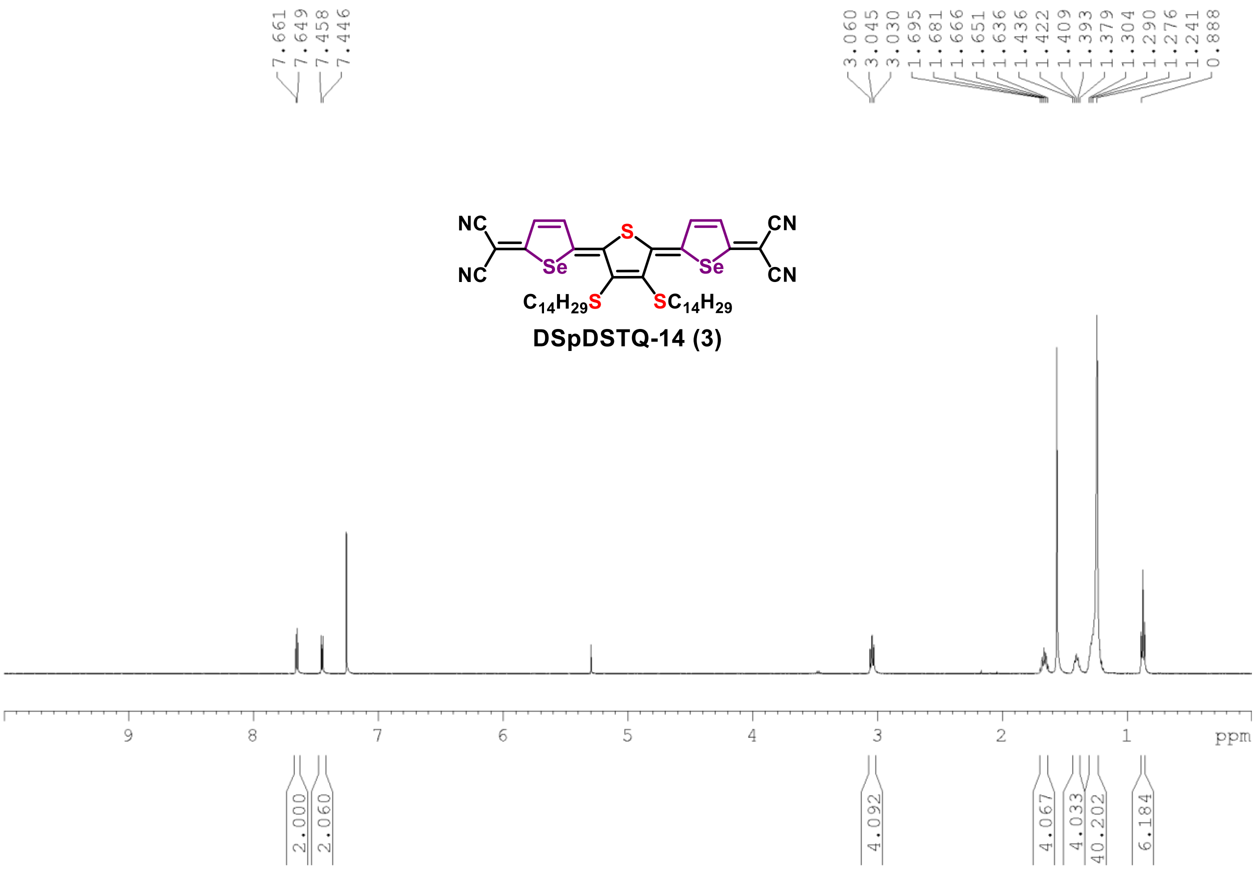
7.661
7.649
7.458
7.446
3.060
3.045
3.030
1.695
1.681
1.666
1.651
1.636
1.436
1.422
1.409
1.393
1.379
1.304
1.290
1.276
1.241
0.888
NC
NC
S
Se
Se
CN
CN
C14H29S
SC14H29
DSpDSTQ-14 (3)
9
8
7
6
5
4
3
2
1
ppm
2.000
2.060
4.092
4.067
4.033
40.202
6.184

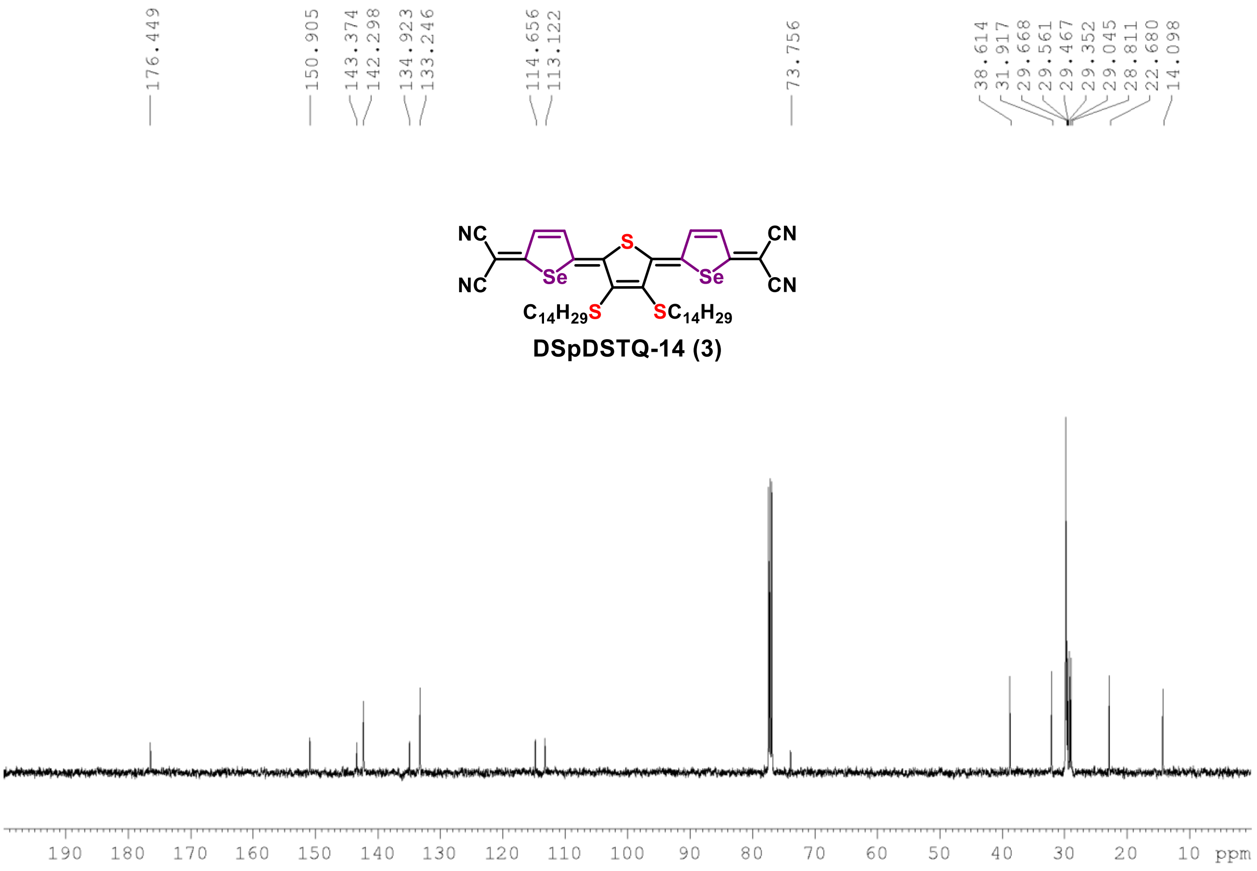
176.449
150.905
143.374
142.298
134.923
133.246
114.656
113.122
73.756
38.614
31.917
29.668
29.561
29.467
29.352
29.045
28.811
22.680
14.098
NC
NC
S
Se
Se
CN
CN
C14H29S
SC14H29
DSpDSTQ-14 (3)
190
180
170
160
150
140
130
120
110
100
90
80
70
60
50
40
30
20
10
ppm

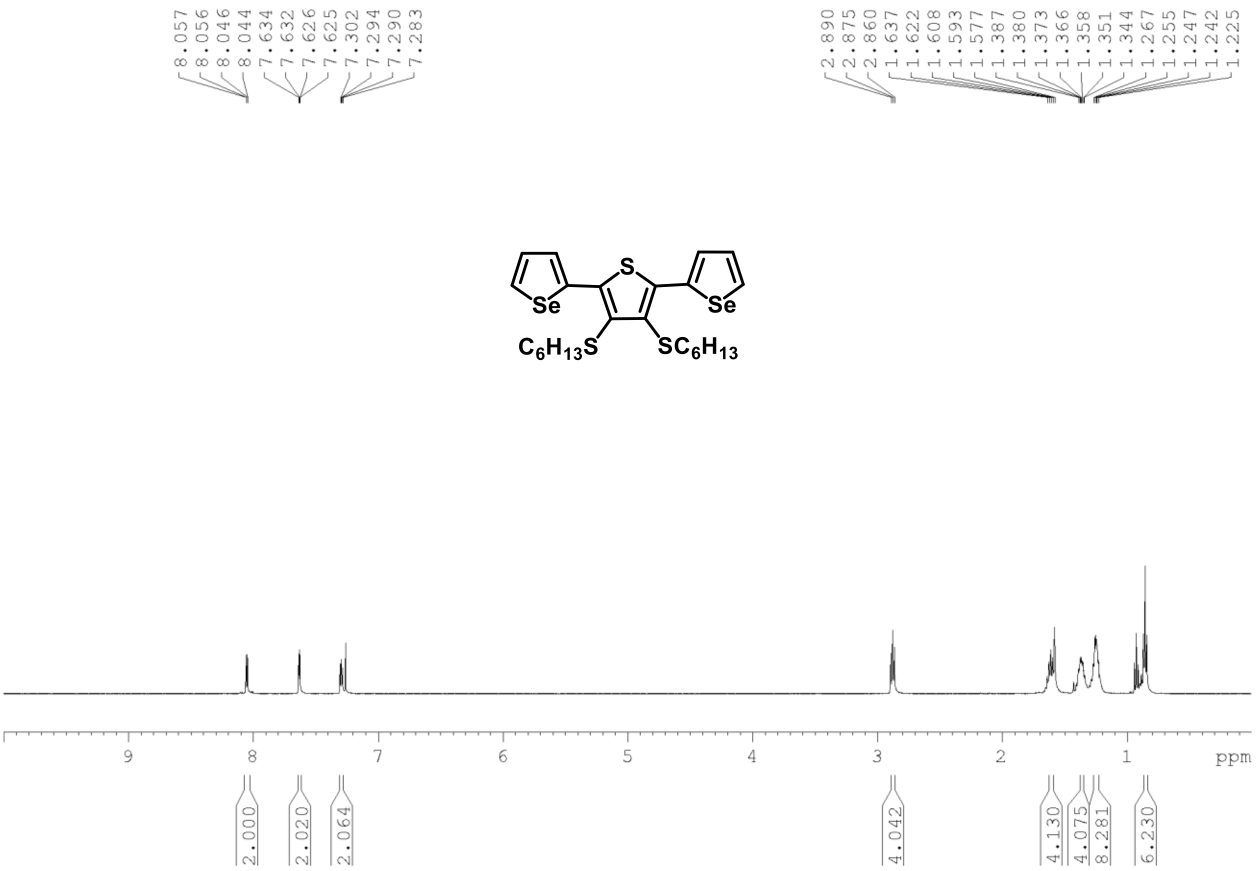
8.057
8.056
8.046
8.044
7.634
7.632
7.626
7.625
7.302
7.294
7.290
7.283
2.890
2.875
2.860
1.637
1.622
1.608
1.593
1.577
1.387
1.380
1.373
1.366
1.358
1.351
1.344
1.267
1.255
1.247
1.242
1.225
S
Se
Se
C6H13S
SC6H13
9 8 7 6 5 4 3 2 1 ppm
2.000
2.020
2.064
4.042
4.130
4.075
8.281
6.230

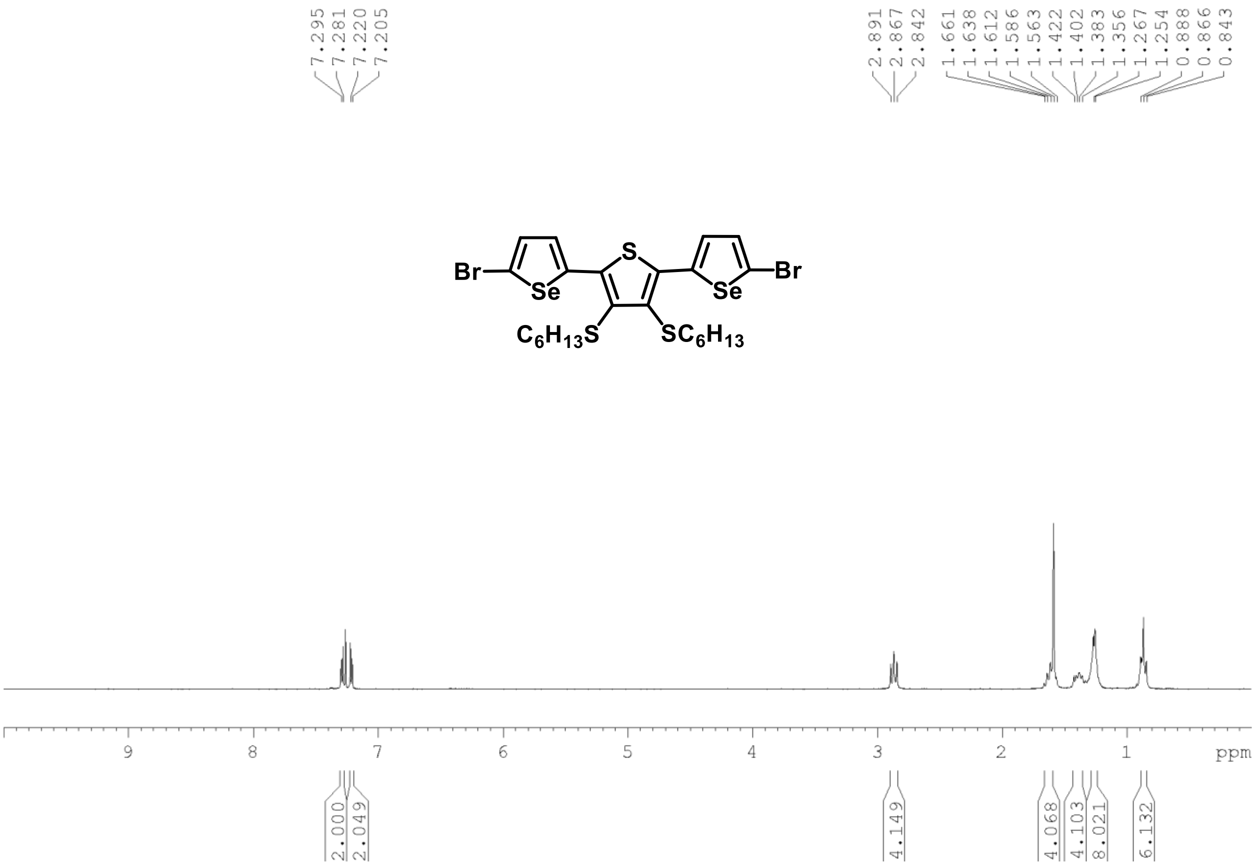
7.295
7.281
7.220
7.205
2.891
2.867
2.842
1.661
1.638
1.612
1.586
1.563
1.422
1.402
1.383
1.356
1.267
1.254
0.888
0.866
0.843
Br
S
Se
Se
Br
C6H13S
SC6H13
9 8 7 6 5 4 3 2 1 ppm
2.000
2.049
4.149
4.068
4.103
8.021
6.132

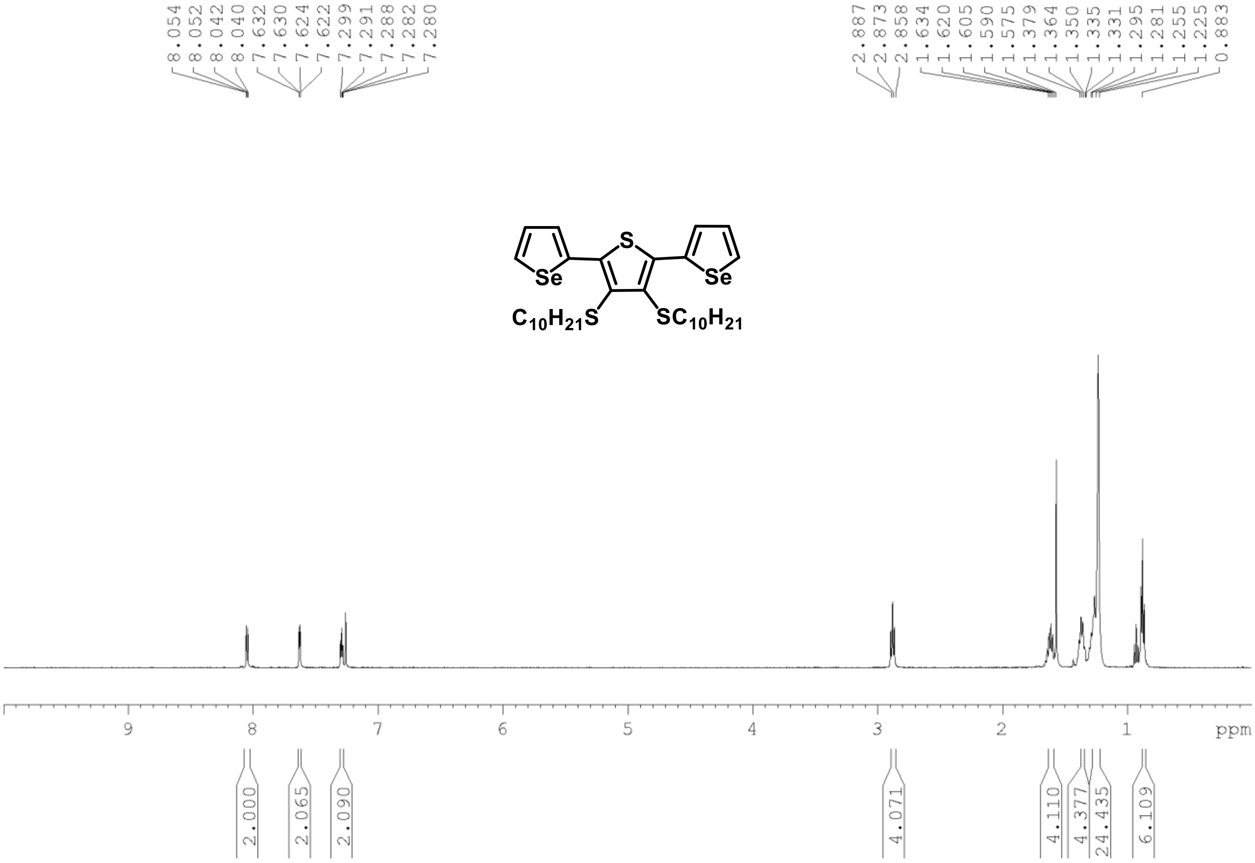
8.054 8.052 8.042 8.040 7.632 7.630 7.624 7.622 7.299 7.291 7.288 7.282 7.280
2.887 2.873 2.858 1.634 1.620 1.605 1.590 1.575 1.379 1.364 1.350 1.335 1.331 1.295 1.281 1.255 1.225 0.883
S
Se
Se
C10H21S
SC10H21
9 8 7 6 5 4 3 2 1 ppm
2.000 2.065 2.090 4.071 4.110 4.377 24.435 6.109

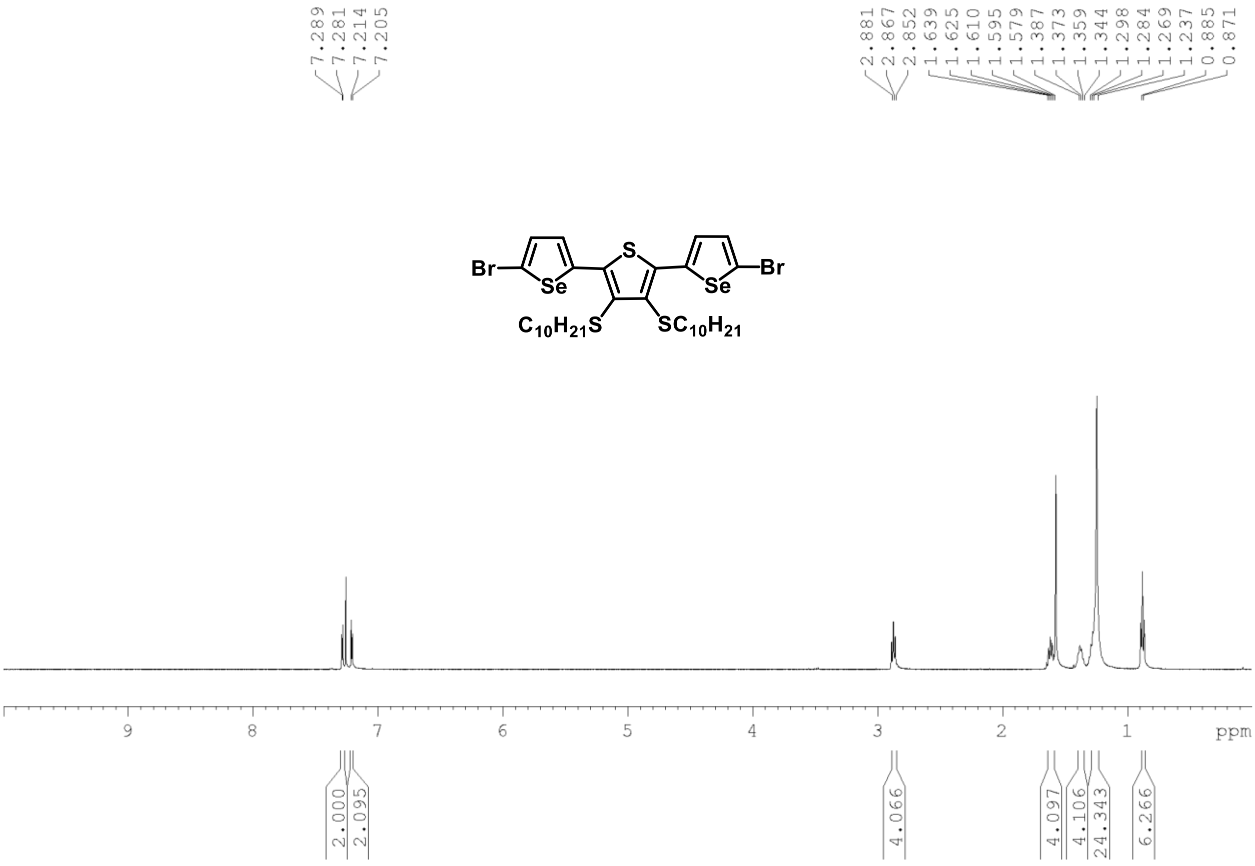
7.289 7.281 7.214 7.205
2.881 2.867 2.852 1.639 1.625 1.610 1.595 1.579 1.387 1.373 1.359 1.344 1.298 1.284 1.269 1.237 0.885 0.871
Br
S
Se
Se
Br
C10H21S
SC10H21
9 8 7 6 5 4 3 2 1 ppm
2.000 2.095 4.066 4.097 4.106 24.343 6.266

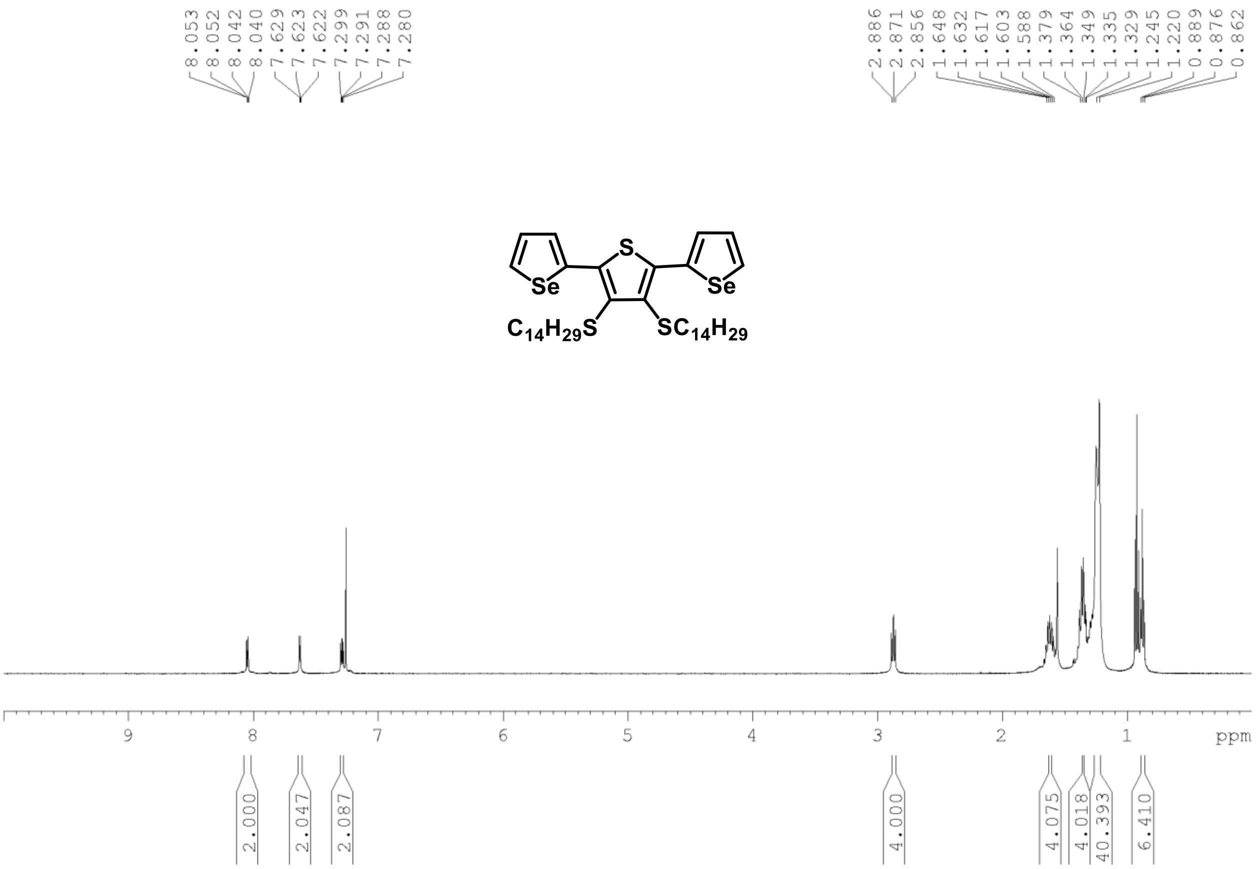
S
Se
Se
C14H29S
SC14H29
ppm

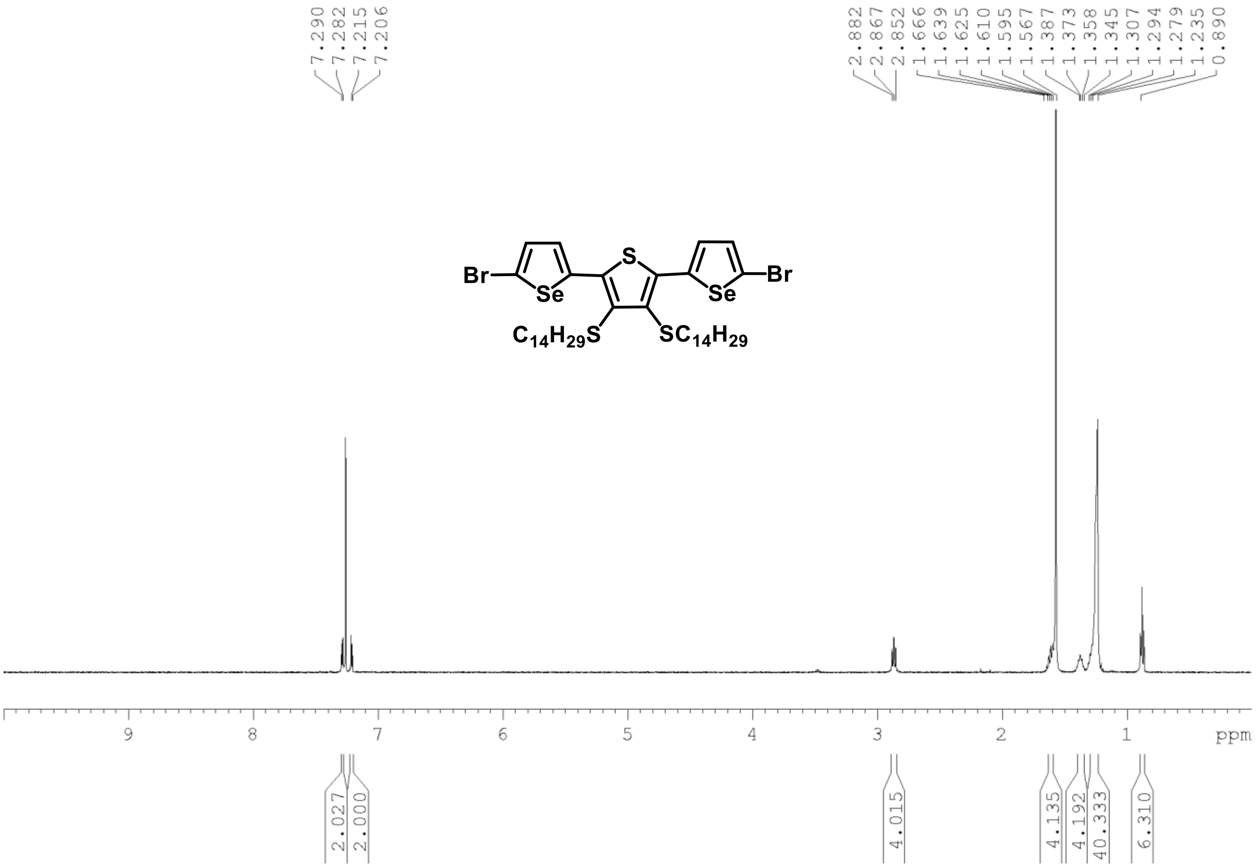
Br
S
Se
Se
Br
C14H29S
SC14H29
ppm

Formula	Mass	Error	mSigma	DblEq	N rule	Electron Configuration
C 30 H 30 N 4 S 3 Se 2	701.9958	1.4900	85.8458	18.00	ok	odd

Comment 1 DSpDSTQ-6_701.9963

Comment 2

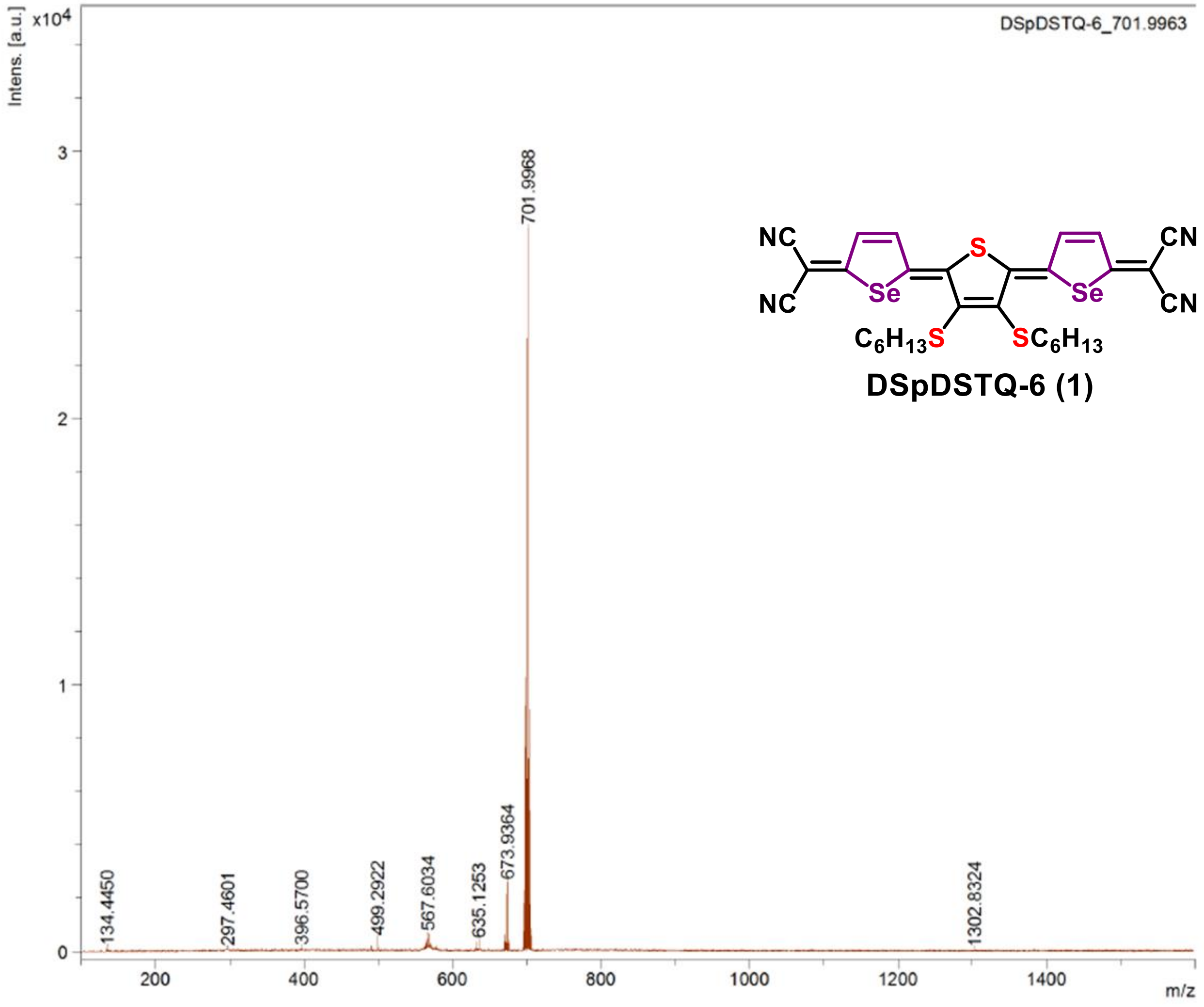

Formula	Mass	Error	mSigma	DblEq	N rule	Electron Configuration
C 38 H 46 N 4 S 3 Se 2	814.1210	0.5914	116.0986	18.00	ok	odd

Comment 1 DSpDSTQ-10_814.1215

Comment 2

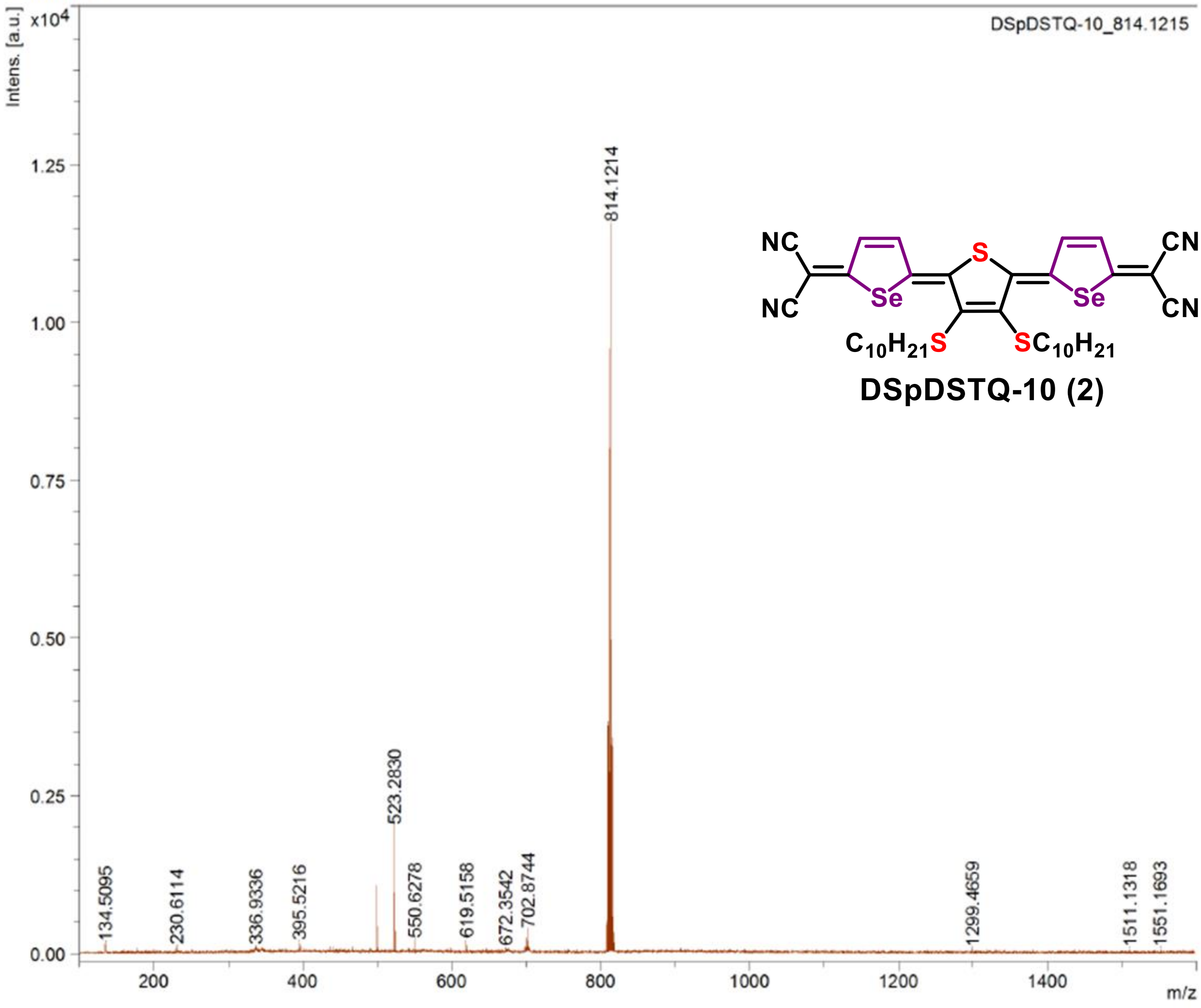

Formula	Mass	Error	mSigma	DblEq	N rule	Electron Configuration
C 46 H 62 N 4 S 3 Se 2	926.2462	2.5317	105.3332	18.00	ok	odd

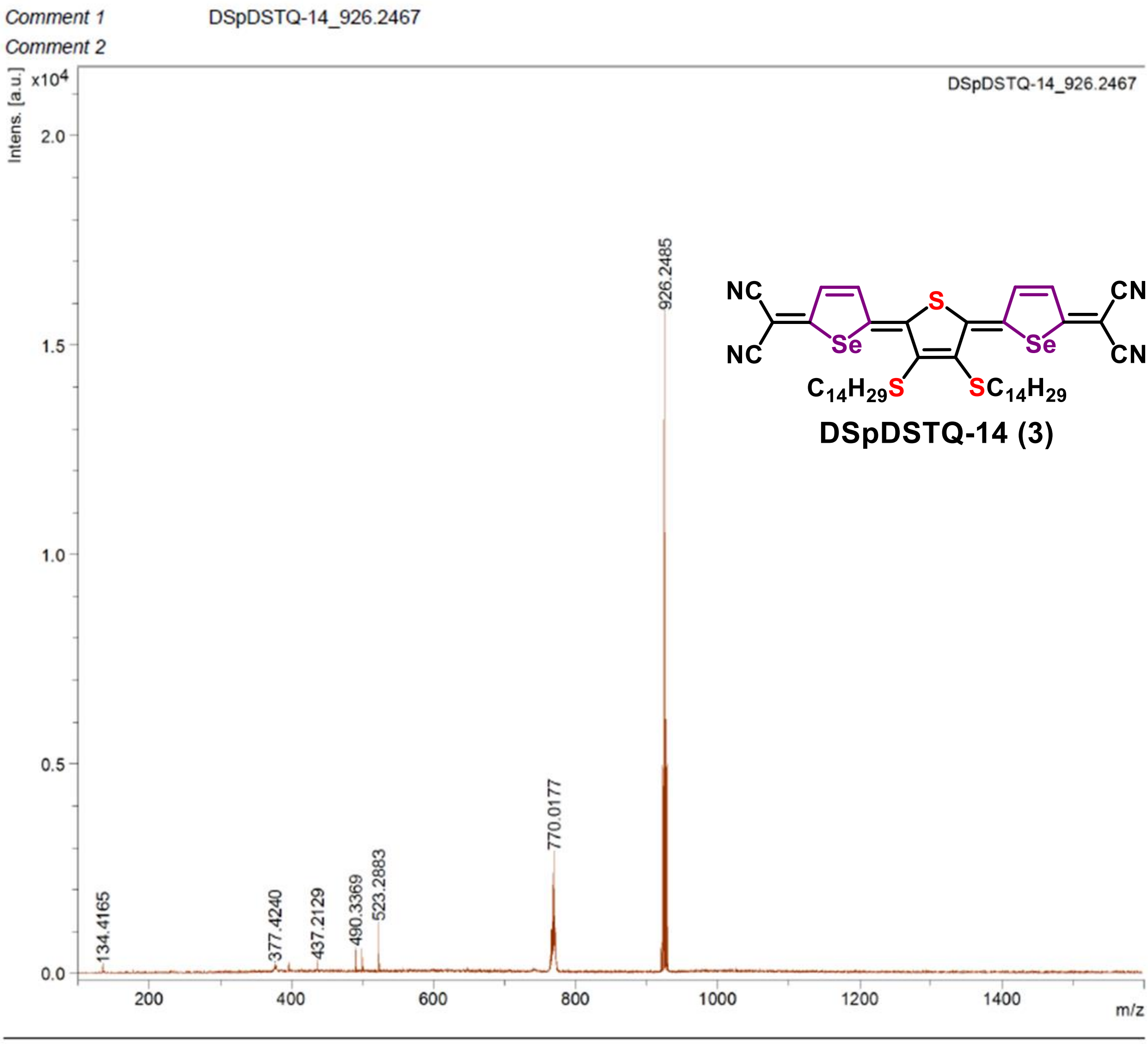

References

[1] S. Vegiraju, A. A. Amelenan Torimtubun, P.-S. Lin, H.-C. Tsai, W.-C. Lien, C.-S. Chen, G.-Y. He, C.-Y. Lin, D. Zheng, Y.-F. Huang, *ACS Appl. Mater. Interfaces* **2020**, *12*, 25081.

[2] B. Kim, H. R. Yeom, M. H. Yun, J. Y. Kim, C. Yang, *Macromolecules* **2012**, *45*, 8658.
